# The dynamic landscape of chromatin accessibility and active regulatory elements in the mediobasal hypothalamus influences the seasonal activation of the reproductive axis in the male quail under long light exposure

**DOI:** 10.1186/s12864-024-10097-5

**Published:** 2024-02-19

**Authors:** Jianye Chang, Yanglong Xu, Yuting Fu, Jiaxin Liu, Danli Jiang, Jianqiu Pan, Hongjia Ouyang, Wenjun Liu, Jin Xu, Yunbo Tian, Yunmao Huang, Jue Ruan, Xu Shen

**Affiliations:** 1 College of Animal Science & Technology, Zhongkai University of Agriculture and Engineering, Guangzhou, 510225 China; 2 Shenzhen Branch, Guangdong Laboratory of Lingnan Modern Agriculture, Genome Analysis Laboratory of the Ministry of Agriculture and Rural Affairs, Agricultural Genomics Institute at Shenzhen, Chinese Academy of Agricultural Sciences, Shenzhen, 518120 China; 3 State Key Laboratory of Biocontrol, School of Life Sciences, Sun Yat-sen University, Guangzhou, 510642 China

**Keywords:** Chromatin accessibility, Transcription factor, Seasonal reproduction, *Coturnix japonica*

## Abstract

**Background:**

In cold and temperate zones, seasonal reproduction plays a crucial role in the survival and reproductive success of species. The photoperiod influences reproductive processes in seasonal breeders through the hypothalamic-pituitary-gonadal (HPG) axis, in which the mediobasal hypothalamus (MBH) serves as the central region responsible for transmitting light information to the endocrine system. However, the cis-regulatory elements and the transcriptional activation mechanisms related to seasonal activation of the reproductive axis in MBH remain largely unclear. In this study, an artificial photoperiod program was used to induce the HPG axis activation in male quails, and we compared changes in chromatin accessibility changes during the seasonal activation of the HPG axis.

**Results:**

Alterations in chromatin accessibility occurred in the mediobasal hypothalamus (MBH) and stabilized at LD7 during the activation of the HPG axis. Most open chromatin regions (OCRs) are enriched mainly in introns and distal intergenic regions. The differentially accessible regions (DARs) showed enrichment of binding motifs of the RFX, NKX, and MEF family of transcription factors that gained-loss accessibility under long-day conditions, while the binding motifs of the nuclear receptor (NR) superfamily and BZIP family gained-open accessibility. Retinoic acid signaling and GTPase-mediated signal transduction are involved in adaptation to long days and maintenance of the HPG axis activation. According to our footprint analysis, three clock-output genes (TEF, DBP, and HLF) and the THRA were the first responders to long days in LD3. THRB, NR3C2, AR, and NR3C1 are the key players associated with the initiation and maintenance of the activation of the HPG axis, which appeared at LD7 and tended to be stable under long-day conditions. By integrating chromatin and the transcriptome, three genes (DIO2, SLC16A2, and PDE6H) involved in thyroid hormone signaling showed differential chromatin accessibility and expression levels during the seasonal activation of the HPG axis. TRPA1, a target of THRB identified by DAP-seq, was sensitive to photoactivation and exhibited differential expression levels between short- and long-day conditions.

**Conclusion:**

Our data suggest that trans effects were the main factors affecting gene expression during the seasonal activation of the HPG axis. This study could lead to further research on the seasonal reproductive behavior of birds, particularly the role of MBH in controlling seasonal reproductive behavior.

**Supplementary Information:**

The online version contains supplementary material available at 10.1186/s12864-024-10097-5.

## Introduction

The reproductive cycle of birds is strictly controlled by day length (photoperiod), which results in profound seasonal differences in reproductive behavior when birds are maintained under long- and short-day photoperiods [1]. In short-daybred Magang geese, initial exposure to long days leads to gonadal inactivity, but switching to short-day conditions drives gonadal activity [2]; however, in long-daybred quail, maintaining short day inhibits gonadal activity, but changing to long-day conditions activates the reproductive axis and promotes the secretion of testosterone [3]. The underlying molecular mechanism governing seasonal reproduction in birds is markedly different from that in mammals. In mammals, photoperiodic information is transduced to melatonin production by the pineal gland. It is the primary driver of the cascade of molecular signals involved in the neuroendocrine control of seasonal rhythms [4]. In birds, melatonin is not necessary to provide the internal physiological code for photoperiod regulation of seasonal rhythms. Light information directly acts on the deep brain photoreceptors of the hypothalamus [5–8]. However, how light activates these photoreceptors and subsequently affects GnRH secretion in the Mediobasal hypothalamus (MBH) is still poorly understood.

The genetic mechanism underlying the seasonal activation of the hypothalamic-pituitary-gonadal axis (HPG) axis is one of the most challenging scientific questions in seasonal reproduction. Previous reports on quail and sheep showed that triiodothyronine (T3) synthesized by Tanycytes from the MBH, is the dominant signal controlling the activation of the reproductive axis [9, 10]. T3 plays an essential role in seasonal activation of the HPG axis. Previous studies have analyzed in detail the photoperiod-driven neuroendocrine mechanism of T3 driven by the photoperiod: the light input path of poultry is different from that of mammals, and the photoperiod information is sensed through the deep brain photoreceptors [5, 6, 11]. Light signals are is transmitted to the pars tuberalis (PT) of the pituitary gland and regulate the seasonal secretion of thyroid stimulating hormone (TSH) by the thyrotropes cells [12, 13]. TSH retrogradely acts on tanycytes in the third ventricle, promoting the expression of DIO2 and regulating the Thyroid Hormone (TH) metabolism by controlling the gene expression of DIO2. DIO2 regulates the conversion of thyroxine (T4) to the more bioactive T3, while DIO3 degrades T4 and T3 to the inactive reverse triiodothyronine (rT3) [9, 14]. T3 can activate gonads that are dormant under short-day conditions and restore them to a reproductive state [9, 15].

The most notable feature of reproductive axis initiation is the timely production and secretion of gonadotropin-releasing hormone (GnRH) in the hypothalamus. However, increased T3 synthesis in the MBH can lead to reproductive inactivity in short-day breeding. Moreover, T3 can induce reproductive activity during long-day breeding [9, 12, 16–18], which suggests that the activation and regulation of T3 on the reproductive axis in long-day and short-day breeding animals exhibit functional differentiation. Long days trigger an increase in the concentration of local T3; Hypothalamic GnRH-I mRNA expression varies in response to photoperiodic conditions [19], and secretion of GnRH follows a circadian pattern; however, how T3 impacts GnRH secretion is still unclear, and the related neuroendocrine pathways, acting cells, and molecular targets involved remain controversial [20, 21].

Seasonal secretion of GnRH is the most significant feature of reproductive axis activation in the hypothalamus. Tanycytes, the most common type of glial cell in the central nervous system of vertebrates [22], are the primary source of T3 in the hypothalamus and are widely believed to be critical for controlling GnRH secretion and reproductive initiation [23]. Tanycytes act as sensors and neuroendocrine output modulators of the HPG axis [24]. Cytological evidence suggests that tanycytes participate in the seasonal release of GnRH through a plastic spatial reconstruction between tanycytes and GnRH neuron terminals [16, 25–28]. This process is highly dependent on reversible intracellular dynamic changes in chromatin structure, chromatin accessibility, and modification [29–32]. Numerous studies have shown that photoperiodic adaptation produces phenotypes (such as rhythm, metabolism, feeding, dormancy, and reproduction) associated with changes in epigenetic markers [30, 31, 33–35].

We speculate that the diversity of the reproductive phenotype is more likely to affect gene expression regulation than to alter protein function. Epigenetic modifications are vital for shaping seasonal reproductive phenotypes. Recent comparative genomic studies on Atlantic herring have shown that a noncoding variant at the TSHR locus may contribute to regulating seasonal reproduction [36]. Resequencing results of the chicken genome revealed that missense mutation of TSHR led to a decrease in the seasonal breeding characteristics of domestic chickens [37, 38]. These findings suggest that epigenetic modification and chromatin remodeling are necessary for the occurrence of correct seasonal reproductive behaviors.

Regulatory elements within accessible chromatin control gene expression. Accumulating evidence has shown that changes in chromatin accessibility are related to seasonal reproduction [16, 32]. To comprehensively elucidate the regulatory mechanisms of seasonal reproduction in birds, we induced the process of reproductive axis activation by modulating the photoperiod in quail. ATAC-seq is a powerful method for identifying functional elements throughout the genome, and it can provide insights into the regulatory mechanisms that underlie changes in the seasonal reproductive activation of the HPG axis.

## Materials and methods

### Artificial photoperiodism and sample collection

The experimental animals and the design of the artificial photoperiodism program have been previously described [3]. Briefly, the artificial photoperiod used to induce the seasonal activation of quails is performed.

A total of 240 male quails, aged 7 weeks, underwent a 4-week prefeeding period with unrestricted access to standard food and water. This process was conducted under controlled conditions at a constant room temperature of 21 °C, with a light intensity of 245 lux, following a light–dark cycle consisting of 16 hours of light and 8 hours of darkness. Four weeks later, the quails were initially exposed to short-day conditions [(6 light (L):18 dark (D)] (6 L:18D) for a duration of 28 days (SD28) and then subjected to long-day conditions (20 L:4D) for the subsequent 28 days to stimulate testis recovery. The inactivation phenotype of the reproductive axis in quail is associated with the testicular morphological and functional alterations. Long days promote the reproductive activity of the quail population, and MBH samples were collected from SD28 plants under long-day conditions at days 3 (LD3), days 7 (LD7), and days 28 (LD28) days (*n* = 6 biologically independent samples). The quails were euthanized by inhalation of carbon dioxide and cervical dislocation, performed by a laboratory technician with extensive experience in applying the techniques. Mediobasal hypothalamus samples were collected and dissected to explore the changes in open chromatin accessibility during the transition from long to short light in quail. All mediobasal hypothalamus tissues were collected at 8 h after lights-on zeitgeber time 8 (ZT8).

### Histological and TEM examination of the mediobasal hypothalamus

The mediobasal hypothalamus was excised rapidly after the bird was euthanized. The brain tissues were initially trimmed to a small 1 mm^3^ size and immediately fixed in glutaraldehyde for 12 h at room temperature. The fixed tissues were embedded in resin. The specimens were dehydrated in graded concentrations of ethanol and acetone. An ultrathin microtome was used to cut the resin blocks into 60–80 nm ultrathin slices. The sections were stained with 150 mesh copper mesh in 2% uranium acetate saturated alcohol solution in the dark for 8 min; Wash with 70% alcohol three times, cleaned with ultrapure water three times, stained with 2.6% lead citrate solution and carbon dioxide for 8 min, cleaned with ultrapure water three times and blotted slightly with filter paper. The copper mesh slices were placed in a copper mesh box and allowed to dry overnight at room temperature.

### Measurement of the local concentration of T3 in the hypothalamus by UPLC–MS//MS

After whole mediobasal hypothalamus homogenization, samples from both SD28 and LD28 were processed for liquid chromatography–mass spectrometry (LS-MS) detection within 24 h. Local T3 was measured via the Acquity-I Xevo UPLC–MS/MS detection platform (Waters Corp., Milford, MA, USA). A Waters ACQUITY UPLC system was used (Waters, USA). Chromatography was performed on an ACQUITY UPLC BEH C18 1.7 μM analytical column (2.1 × 100 mm, Waters, USA). A total of 7.5 μL of each sample was loaded with the autosampler, and the column temperature was 40 °C. Under mass spectrometry conditions, a positive ion model was used with the following parameters: ion source temperature = 500 °C; ion spray voltage = 5000 V; curtain gas (nitrogen) = 30 psi; and both the atomizing gas and auxiliary gas = 60 psi.

### ATAC-seq library preparation and data analysis

Nuclei were isolated from the frozen mediobasal hypothalamus according to the protocol of Ryan Corces [39]. The frozen pituitary was placed into a prechilled 2 mL Dounce tissue grinder set (Cat. No. D8938-1SET) (Sigma–Aldrich, Darmstadt, Germany) containing 2 mL cold 1x HB and then thawed for 5 min. The tissue was filtered during transfer using a 70 μm cell strainer (Cat. No. 431751) (Corning, New York, USA), and the homogenate was transferred to a prechilled Eppendorf 2 mL Lo-Bind tube (Cat. No. Z666556-250EA) (Sigma–Aldrich, Darmstadt, Germany). Pellet nuclei were generated by spinning the homogenate for 5 min at 4 °C at 350 RCF in a fixed-angle centrifuge. Iodixanol (Cat. No. D1556–250 mL) (Sigma-Aldrich, Darmstadt, Germany) was used for collecting the nuclei. The isolated were counted via trypan blue staining on a cell counter, and 30 K high-quality nuclei were used to prepare an ATAC-seq library.

This ATAC-seq library was prepared following the instructions of the TruePrep DNA Library Prep Kit V2 for Illumina (Vazyme, Nanjing, China). Nuclei pellets were incubated in a 50 μL of transposition mix (10 μL of 5x TTBL buffer, 5 μL of TTE Mix, and 35 μL of ddH_2_O) for 30 min at 37 °C. The transposed DNA was then purified with VAHTS DNA cleaning beads (Vazyme, Nanjing, China) and amplified for 12 ~ 15 cycles via PCR. Libraries were purified and assessed for fragment length distribution with a Bioanalyzer Qseq 100 Bio-Fragment Analyzer (Bioptic Inc.) and subjected to paired-end 150 bp sequencing on the NextSeq platform.

Fastp [40] software was used to remove adapter sequences, poly-X at the 3′ end and reads with a phred-scaled quality score of less than 15 for more than 15% of the bases. The trimmed fastq files were mapped to the *Coturnix japonica* 2.1 genome obtained from the NCBI database (*Coturnix japonica* 2.1, GCA_001577835.2) using Bowtie2 [41] with the name ‘--very-sensitive -X 2000’. The resultant sequence alignment map (SAM) files were compressed to the binary alignment map (BAM) version, on which SAMtools [42] was used to filter reads that were unmapped, mate unmapped, not primary alignment, or failed platform quality checks. The read pairs mapped to mitochondrial DNA were discarded using SAMtools. Redundant read pairs from PCR amplification were removed afterward using Picard MarkDuplicates [43]. The filtered bam files were subsequently converted into normalized bigwig files using bedtools [44] to visualize the peaks. The deepTools package [45] was used to move the positive chain of the BAM file forward by 4 bp and the negative chain negative by 5 bp with the parameter ‘--ATACshift’. Then, open accessible regions for each shifted bam file were defined by the peaks identified by MACS2 [46] with the parameters “-g ‘genome size’ -f BAMPE –q 0.05 --keep-dup all”. Specifically, the commonly shared peaks according to multiple biological replicates for each time point were first obtained by bedops [47]. The four pints’ shared peaks were merged and retained for the following analysis. Bedtools was also used to calculate the read coverage of the above peak regions. All the statistical analyses were performed by pairwise comparisons across every time point. Analysis of ATAC-seq data for differential accessibility was carried out using negative binomial regression with normalization based on the size factors in the DESEQ2 package [48] with the thresholds of | log2FC| > = 0.8 and *P* < = 0.05. The K-means algorithm was subsequently used cluster all differential peaks in R. The Gene Ontology (GO) enrichment analysis of genes annotated by differential peaks was subsequently performed using Metascape (https://metascape.org/) [49]. The HOMER Field 102 function findMotifsGenome.pl was used to perform motif analysis of the peak regions. Computational footprint analysis was conducted across each merged bam file using TOBIAS [50], default parameter settings, and the *Coturnix japonica* 2.1 genome. The annotations of the peaks were achieved using ChIPseeker [51] in R. ATAC-seq peak visualization was generated using IGV [52].

### RNA-seq library preparation and data analysis

Total RNA from the frozen mediobasal hypothalamus was subjected to transcriptome sequencing on an Illumina HiSeq 4000 platform (Guangzhou, China) to generate 150 bp paired-end reads. The RNA-seq raw fastq data were quality controlled, and the reads were trimmed by filtering with Fastp. The clean reads were subsequently aligned to the *Coturnix japonica* 2.1 reference genome using the STAR [53] with the default parameters. FeatureCounts [54] and the corresponding genome annotation file (GTF file) from NCBI were used to estimate transcript abundance and generate FPKM values. The DEGs were analyzed by the DESEQ2 package in R under the criteria of a |log2- fold change (FC)| > = 1 and a false discovery rate (FDR) < = 0.05. The Gene Ontology (GO) enrichment analysis of the differentially expressed genes was performed using Metascape. For published transcriptome online data, we used the same analysis pipeline as that used for our data.

### DAP-seq library preparation and data analysis

After extracting the DAP DNA, the enriched DNAs were fragmented into short fragments by ultrasonic. Next, the DNA fragments were end-repaired, 3’A was added, and the sequencing adapters was ligated to the Illumina platform. DNA fragments of the proper size were selected for PCR amplification. Finally, we obtained a qualified library for sequencing. The samples were PCR amplified and sequenced using an IlluminaHiSeqTM 4000 by Gene Denovo Biotechnology Co. (Guangzhou, China). The reads obtained from the sequencing machines included raw reads containing adapters or low-quality bases, which affected the subsequent assembly and analysis. The raw reads were processed to obtain high-quality clean reads according to three stringent filtering standards: (1) reads containing adapters were removed; (2) reads containing more than 10% of unknown nucleotides (N) were removed; (3) low-quality reads containing more than 40% of low- quality (Q value≤10) bases were removed. Bowtie 2 was used to align the clean reads from each sample against the reference genome. All reads from the transcriptional initiation site (TSS) to the transcriptional termination site (TES) interval and upstream and downstream 2 k interval were counted by deepTools software. MACS2 software was used to identify read-enriched regions from the DAP-seq data. The dynamic Poisson distribution was used to calculate the *p* value of the specific region based on the unique mapped reads. A region was defined as a peak when the q value < 0.05. Peak-related genes were annotated by the ChIPseeker R package. All the DAP-seq peak-related genes were mapped to GO terms in the Gene Ontology database (http://www.geneontology.org/), gene numbers were calculated for every term, and significantly enriched GO terms in peak-related genes compared to the genome background were defined by hypergeometric tests. The MEME suite [22] (http://meme-suite.org/) was used to detect the motifs. MEME (http://meme-suite.org/tools/meme) and DREME (http://memesuite.org/tools/dreme) were used to detect the sequence motif, which was determined to detect long and short consensus sequences.

## Results

### Changes in ultrastructural and local thyroid hormone levels in the mediobasal hypothalamus during seasonal activation of the HPG axis

**Fig. 1:**
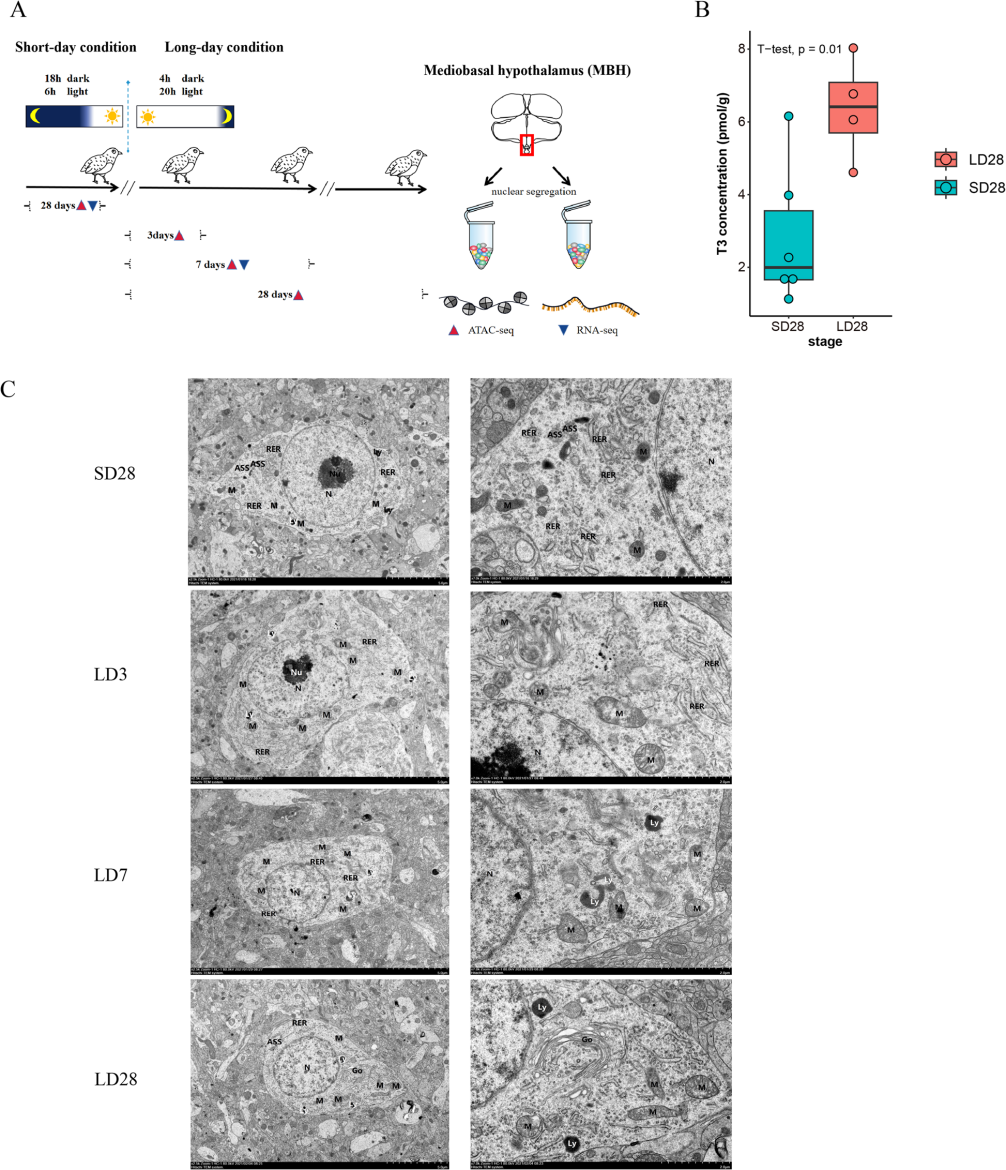
Schematic experimental design of an artificial photoperiod program performed to induce the activation of the HPG axis in male quails. The mediobasal hypothalamic tissues of male Japanese quail were collected under SD28, LD3, LD7, and LD28 conditions, and accessible chromatin was detected by high throughput sequencing (ATAC-seq). Tissues were collected on SD28 and LD7 conditions for RNA sequencing (RNA-seq). SD28, feed for 28 days under short-day conditions (6 h light, 18 h dark); LD3, continued providing for 3 days after conversion to long light conditions (20 h light, 4 h dark); LD7, continued feeding for 7 days after conversion to long light conditions; LD28, continued feeding for 28 days after conversion to long-day conditions. **A** Induction of the seasonal activation of the HPG axis by artificial photoperiod in quails. **B** Local T3 concentration of mediobasal hypothalamic between quails maintains underlying SD28 and LD28. **C **Transmission Electron microscopic images of the median hypothalamic eminence of male quail during four stages of HPG axis activity. (SD28 corresponds to 28 days under short-day conditions. LD3, LD7, and LD28 correspond to different days of conversion to long-day conditions, respectively)

An artificial photoperiod program was used to induce seasonal activation of the HPG axis in male quail (Fig. 1A). Local T3 concentrations in the mediobasal hypothalamic were detected in quails under short-day and long-day conditions by using UPLC-MS/MS. Compared to that in samples maintained under short-day conditions, a high level of T3 in the mediobasal hypothalamus was observed in the LD28 population (*P* < 0.01) (Fig. 1B). These finding suggested the successful activation of the HPG axis in male quails. Transmission electron microscopy (TEM) was used to reveal the ultrastructural organization of cells in the mediobasal hypothalamus of male quail during the four stages of HPG axis activity (Fig. 1C). The typical autophagic structure of cells can be observed in both SD28 and LD3. The nuclear chromatin patterns of SD28 and LD28 cells differed. The cell’s nuclear membrane in SD28 appeared completely intact and smooth, with a heterochromatin formation. LD3, LD7, and LD28 cells had slightly dented nuclei, fuzzy nuclear membranes, and uniform chromatin. Cells in the LD28 group had transparent nuclear membranes and homogeneous chromatin.

### Mapping the landscape of the chromatin in the mediobasal hypothalamus of quail

**Fig. 2:**
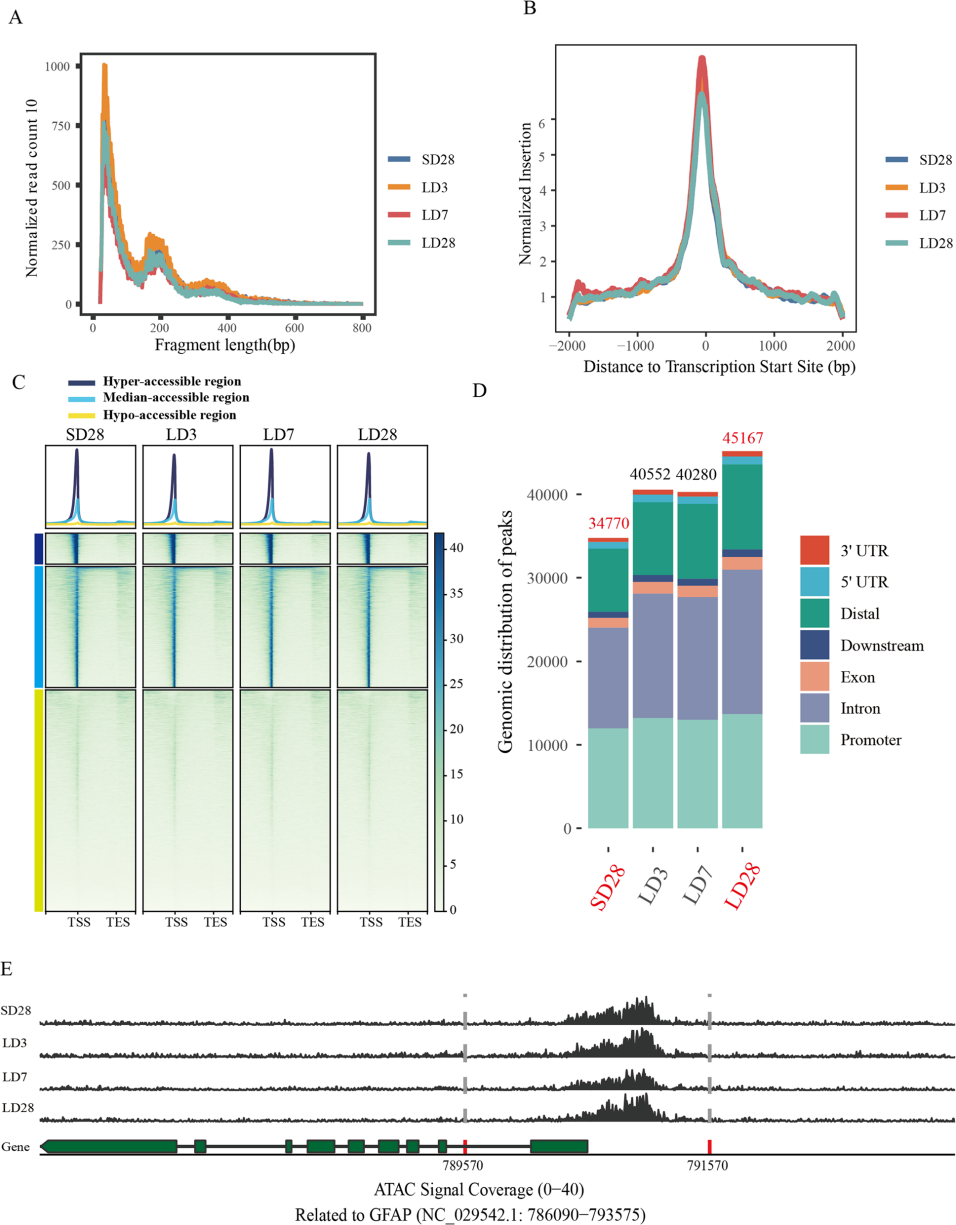
ATAC-seq quality control and genome-wide chromatin accessibility landscape during the activation of the HPG axis. **A** The insert fragments length distribution of ATAC-seq data showed a typical pattern. **B** The fragment size distribution plot indicated that reads were enriched near the transcription start site (TSS). **C** Heatmaps showed the distribution of reads near TSS and TES (2.5 kb before TSS, 1.5 kb after TES), indicating high enrichment near TSS and slight enrichment in TES, and the clustering of open activity of genome-wide genes at different time points (Hyper-accessible region, Medium-accessible region, Hypo-accessible region). **D** statistics of the number of repeatable peaks and genomic location annotation at different time points. **E** The genetic structure of GFAP (green) and chromatin accessibility track at different time points (black), the 2 kb before and after the TSS are marked with red and gray dotted lines. NC_029542.1:786090–793,575 represents the range of visualized tracks

Each ATAC-seq library generated more than 59 million sequencing reads; the Q20, Q30, and GC contents were 93.5 to 98.3%, 86.7 to 94.5%, and 46.26 to 51.99%, respectively. A total of 94.6 to 96.2% of the reads were aligned to the Japanese quail genome from the NBCI. Insert fragment length distributions of mediobasal hypothalamic ATAC-seq data revealed clear nucleosome patterns (Fig. 2A). The distribution and heatmaps of the transcription start site (TSS) enrichment scores for the ATAC-seq sequencing data across the four-time points indicated that the signal around the TSS was more robust (Fig. 2B), indicating that the majority of the identified accessible areas were enriched within 2 kb of the TSS. In addition, a small number of reads were distributed around the transcription termination site (TTS). Heatmap showing the genome-wide distribution of the signal intensity of the differential open chromatin peaks and clustering of the peaks into three clusters, hyperaccessible regions, median accessible regions, and hypoaccessible regions in the mediobasal hypothalamus of quail (Fig. 2C). The expression and secretion of GnRH are recognized as the key marker of the seasonal activation of the HPG axis in birds. It has been reported that the release of GnRH at the GnRH terminal may be regulated by glial cells [55]. Therefore, we examined the chromatin accessibility of the GFAP gene, a signature gene of astrocytes (Fig. 2E), and found an open chromatin region of approximately 600 bp before the TSS of this gene. This result indicated that the gene’s promoter was regulated, suggesting the authenticity of our data. Moreover, we found that technical replicates had a greater Pearson correlation coefficient than did biological replicates .

### Genome-wide chromatin accessibility landscape during seasonal activation of the HPG axis

To explore the accessibility of chromatin across the whole genome, peaks from different replicates of the same experimental treatment were combined, and the regions with high overlap of open chromatin regions (OCRs) were selected for downstream analysis in each treatment group. We detected an average of 34,770, 40,552, 40,280, and 45,167 OCRs at SD28, LD3, LD7, and LD28 respectively, which were used for downstream analysis (Fig. 2D). By visualizing OCRs within the genome, we found that OCRs with higher gene density always have significantly higher chromatin accessibility, consistent with the finding of previous studies. To predict the genomic features of functional regulatory elements, the merged OCRs were annotated, and most of the identified peaks were distributed throughout the genome in the distal intergenic, intronic, and promoter regions (within ±2 kb of the TSS at the transcription start site). The distribution did not change significantly during seasonal activation of the HPG axis (Fig. 2D). Among the four groups, the smallest number of OCRs was observed at SD28, and the total number of peaks increased during the seasonal activation of the HPG axis, which implied that the transcription events increased during this process.

By integrating known TF motifs with our data on reproducible peaks within each group, we obtained the critical transcription factors (*P* value calculated by HOMER <= 10^−90^) that play an important regulatory roles in the process of HPG axis activation. Therefore, we statistically analyzed the sequence within all reproducible peaks at each treatment using HOMER software. The RFX family, CTCF, BORIS, and X-BOX transcription factors were highly enriched. These transcription factors may play an essential roles in maintaining the physical activity of the mediobasal hypothalamus, but there were no significant difference in transcription factors level among the four groups.

### Changes in chromatin accessibility patterns in the MBH during seasonal activation of the HPG axis

To determine how chromatin accessibility changes during seasonal activation of the HPG axis, we accessed the quantitative differences among the four stages. A total of 833 significant DARs were identified by pairwise comparison between the peaks in each group (log2FoldChange > = 0.8, q value <= 0.05). DAR regions were assigned to 693 genes based on the TSS distance to the nearest gene. Compared to those in SD28, we identified 32 DARs in LD3, 210 DARs in LD7, and 509 DARs in LD28 were identified. To evaluate whether chromatin accessibility changes are sustained over time following a long light stimulation, we compared the DARs between two comparison groups: LD3 and LD7 and between LD7 and LD28. Twenty-two DARs were identified between LD3 and LD7. Notably, only 2 DARs were present between LD7 and LD28. These finding suggested that chromatin accessibility status in the mediobasal hypothalamus stabilized in the MBH after 7 days of long light exposure during seasonal activation of the HPG axis.

**Fig. 3:**
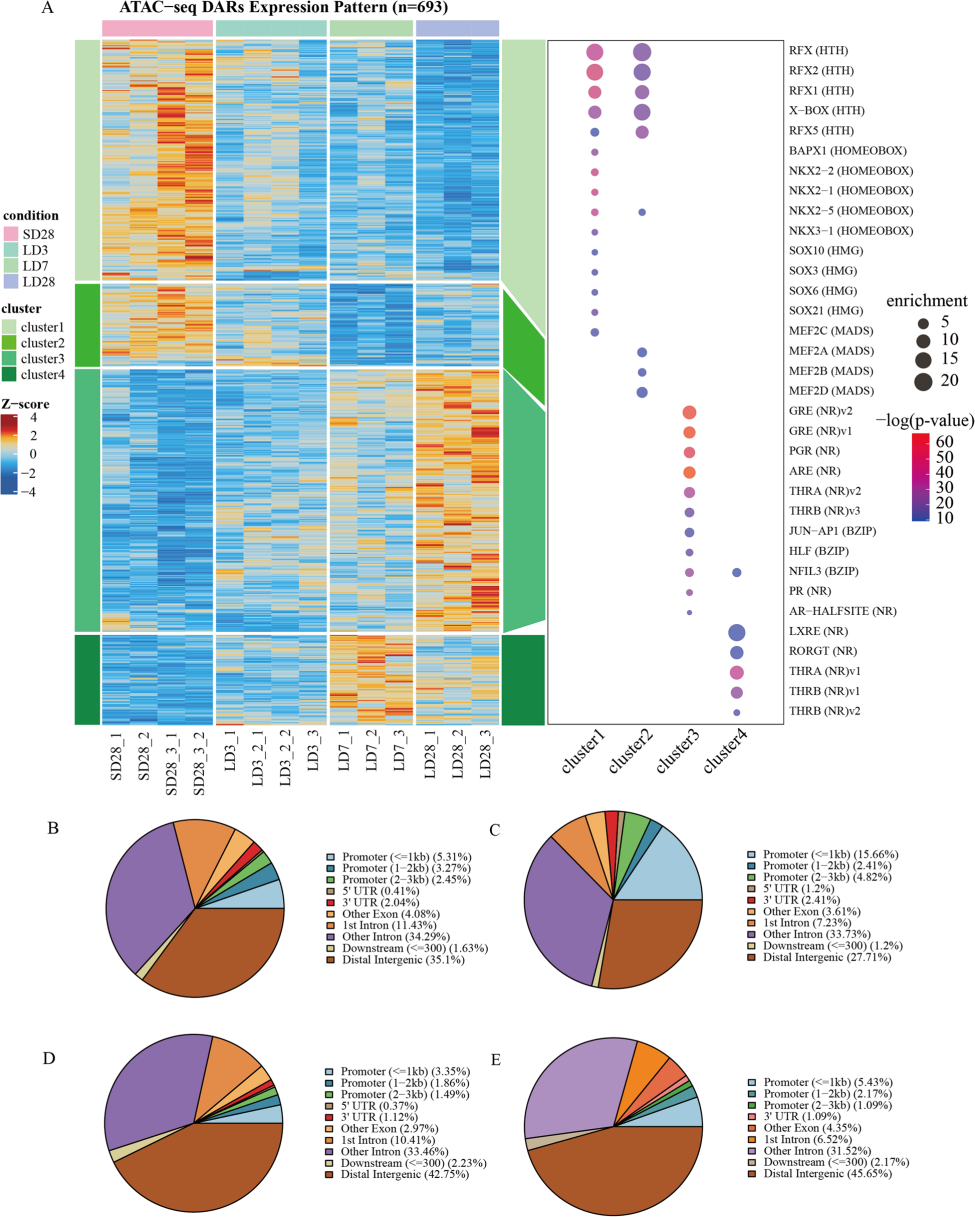
Cluster analysis revealed different patterns of chromatin accessibility and corresponding transcription factors in the hypothalamus during the activation of the HPG axis. **A** In the heat map on the left, each row represents a differential open area, and each column represents an ATAC-seq library sample. Libraries from the same treatment are divided by gaps. Z-scores are normalized area coverage counts, with blue indicating low openness and red indicating high openness. In the bubble diagram on the right, the motif corresponding to each cluster enrichment corresponds to transcription factors, where the bubble size represents enrichment degree (target/background), and the color represents enrichment significance -log (*P*-value). **B** The genomic location annotation of the peak in Cluster 1. **C** The genomic location annotation of the peak in Cluster 2. **D** The genomic location annotation of the peak in Cluster 3. **E** The genomic location annotation of the peak in Cluster 4

To reveal the time-dependent module of chromatin accessibility in the MBH during the HPG axis seasonal activation, we divided all the differentially accessible regions into four clusters by using the k-means method (Fig. 3A). During the process of photoperiodic transformation, different accessibility statutes were present in the mediobasal hypothalamus. Cluster K1 consisted of 246 DARs that exhibited higher accessibility at SD28 and became loss-accessible after prolonged light exposure. Similarly, Cluster K2 included 83 DARs, which presented relatively more accessible chromatin under short-day and turned to gain a loss-accessibility state under long-day conditions. Although both the K1 and K2 clusters were highly open in the short-day stage, Cluster K2 lost accessibility at LD7, while Cluster K1 lost the accessibility at LD28. A greater proportion of DARs in Cluster K1 were mostly located in the promoter region (Fig. 3B-E), which indicated that DARs in Cluster K1 have a tendency to regulate gene transcription. Compared to the loss of chromatin accessibility in SD28, Cluster K3, which comprised 269 DARs, gained chromatin accessibility at LD28, while Cluster K4, which contained 92 DARs, gained chromatin accessibility at the LD7.

To explore the transcription factors that may interact with the peaks of each cluster, we performed motif enrichment analysis of DARs (Fig. 3A). Altered accessibility of transcription factors was observed during the activation of the HPG axis. The motifs of the RFX, NKX, MEF, SOX families, and X-box were enriched in open DARs under short-day conditions, in Cluster K1 and Cluster K2. However, significant enrichment of the binding motifs of both the NR family (GRE, PGR, ARE, THRA, THRB, PR, and AR-Halfsite) and the BZIP family (Jun-AP1, HLF, and NFIL3) was detected in the open DARs under long-day conditions in cluster K3; at the same time, the transcription factors in the NR family were also noted in Cluster K4, including LXRE, RORGT, THRA, and THRB. Among the motifs enriched in open DARs under long-day conditions, motifs of two thyroid hormone receptors, THRA and THRB, were significantly enriched in LD7 and LD28. The enrichment of these motifs may imply their important role in the initiation and maintenance of HPG axis activation induced by long light exposure.

To predict the potential function of each cluster, we further annotated the regions within each cluster to the nearest TSS and performed Gene Ontology (GO) analysis on the annotated genes. The genes near DARs in Cluster K1 were highly enriched in the chromatin binding, regulation of ion transmembrane transport, gated channel activity, axon, and brain development terms. The genes in Cluster K2 were significantly enriched in neuron projection development, glutamatergic synapses, neuron-to-neuron synapses, and GPI-linked ephrin receptor activity. All these biological processes were highly relevant to the functionality of neuron and synapse communication in the MBH under short-day conditions. However, genes in Cluster K3 were significantly enriched in the regulation of small GTPase-mediated signal transduction, regulation of GTPase activity, GTPase activator activity, and GTPase regulator activity. The results for Cluster K3 suggest that there may be a GTPase cascade reaction in response to photoperiod changes at the early stage of the photoperiod transition. In addition, terms related to the cellular response to retinoic acid, sensory organ morphogenesis, inorganic ion homeostasis, G protein-coupled receptor binding, signaling receptor activator activity, and DNA-binding transcription activator activity were highly enriched in Cluster K4. These results imply that retinoic acid signaling and GTPase-mediated signal transduction are involved in adaptation to long days and maintenance of HPG axis activation under long-day conditions.

### Footprint analysis of DARs revealed the critical cis-elements and relevant TFs involved in the activation of the HPG axis

**Fig. 4:**
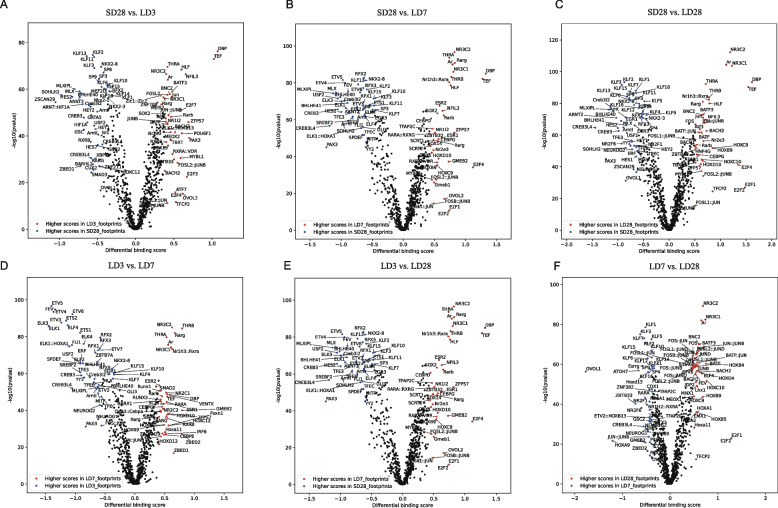
Footprint analysis of ATAC-seq data at different time points. Pairwise comparison of TF activity between different stages. The volcano plots show the differential binding activity against the -log10(P-value) (both provided by TOBIAS) of all investigated TF motifs; each dot represents one motif. **A** Pairwise comparison of TF activity between SD28 and LD3. **B** Pairwise comparison of TF activity between SD28 and LD7. **C** Pairwise comparison of TF activity between SD28 and LD28. **D** Pairwise comparison of TF activity between LD3 and LD7. **E** Pairwise comparison of TF activity between LD3 and LD28. **F** Pairwise comparison of TF activity between LD7 and LD28

The enrichment of TF binding motifs in DARs that gained and lost accessibility during the activation of the HPG axis was investigated. We combined DARs between differential ATAC-seq analyses and performed motif difference-binding analyses based on library timelines across the four time points. By combining these findings with the differences observed via footprint analysis, TFs with a top 5% score and a -log10 (*P*-value) greater than the 95% quantile are highlighted in the volcano plot (Fig. 4A-F).

Compared to those in all the stages under long light stimulation, the footprint of the KLF family of transcription factors was significantly higher in DARs under the short-day conditions. Notably, we identified the binding motifs of many important thyroid hormone signaling TFs during the activation of the HPG axis. Three clock-output genes, thyrotroph embryonic factor (TEF), D element-binding protein (DBP), and hepatic leukemia factor (HLF), exhibited a significantly higher footprint in DARs under the long days. However, these three circadian rhythm-related transcription factors significantly differed between LD3 and LD28. However, there were no differences in binding scores between the LD3 and LD7 comparison groups or between the LD7 and LD28 comparison groups. These results suggest that TEF, DBP, and HLF are the first responders to long -light stimulation and play a sustained regulatory role in the long-day signaling.

Among the long-day response transcription factors, THRA was highly active at the LD3, while the THRB was strongly expressed at LD7. This pattern confirmed that the chromatin accessibility of THR target sites is increased during long-day photoperiods, suggesting that the THRs play a critical role in initial and maintenance of the activation of the HPG axis. Additionally, compared with those under short-day conditions, the footprints of AR, NR3C1, and NR3C2 were significantly increased in DARs, suggesting that these transcription factors may be activated after clock-output genes respond to photoperiodic changes and tend to stabilize their regulatory function under long-day conditions.

### Changes in the transcriptome of mediobasal hypothalamus during seasonal activation of the HPG axis

**Fig. 5:**
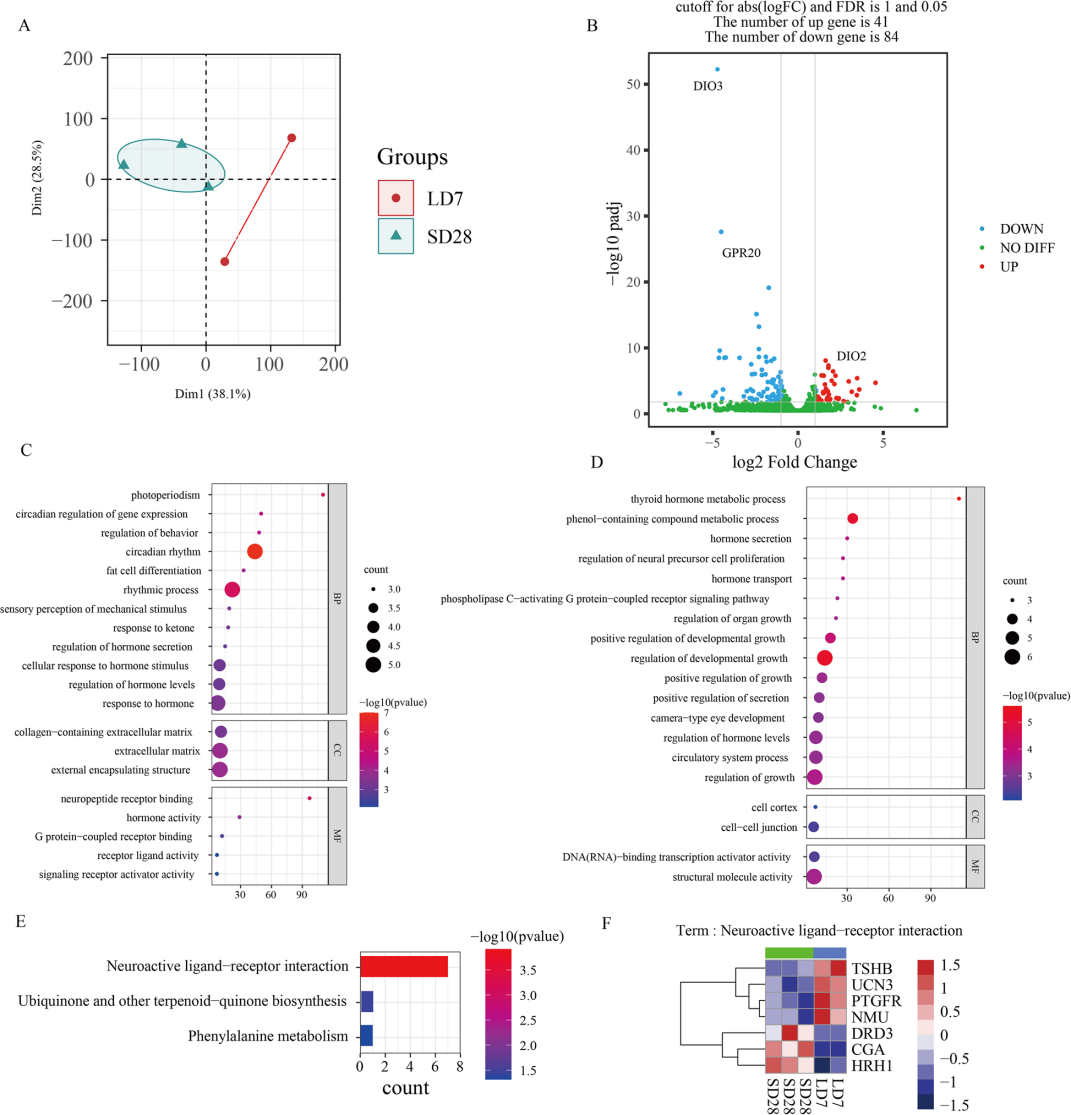
Analysis of differential genes between SD28 and LD7. **A** Principal component analysis of five transcriptome libraries in two states. **B** Visualization of gene expression changes; the ordinate using -log10 p-adj displays the p-adjust value, and the abscissa using log2FoldChange (log2FC) shows the gene expression level. Blue dots indicate the distribution of downregulated DEGs, red dots indicate the distribution of upregulated DEGs, and green dots represent no significant change. **C**-**D** GO function analysis of up-regulated and down-regulated genes, respectively. **E** KEGG pathway analysis of all differential genes. **F** Expression heatmap of the term ‘Neuroactive ligand−receptor interaction’ - associated genes

We performed transcriptome sequencing of the mediobasal hypothalamus regions at SD28 and LD7 because the chromatin accessibility status of LD7 was stable after 7 days of long-light exposure according to the ATAC-seq data. For Each library, more than 8.2 billion clean bases and 27.4 million clean reads were obtained. The Q20 ratio, Q30 ratio, and GC content were 97.46–98, 98, 93.27–94.62, and 50.26–51.23%, respectively. A total of 95.2–95.9% of each library was mapped to the reference Japanese quail genome. PCA of the transcriptome showed a significant difference in the expression patterns between SD28 and LD7 (Fig. 5A). We identified 125 significantly differentially expressed genes (DEGs) (log2fold change> = 1, FDR < =0.05), 41 of which were upregulated and 84 of which were downregulated. These genes may play a critical role in the cascade that activates the photoperiodic responses, including DIO3 and GPR20 downregulation and DIO2 upregulation (Fig. 5B). GO enrichment analysis was used to analyze the upregulated and downregulated genes to reveal the potential biological functions of the DEGs during early photoperiod changes. The GO terms associated with the upregulated genes were mainly enriched in photoperiodic phenomena, circadian regulation of gene expression, and neuropeptide receptor binding. In contrast, the downregulated GO terms were enriched in the thyroid hormone metabolic process (Fig. 5C-D). The KEGG pathway annotation analysis results for all the DEGs were consistent with the GO results, and the term neuroactive ligand receptor interaction was highly enriched, these genes included including TSHB, UCN3, PTGFR, NMU, DRD3, CGA, and HRH1 (Fig. 5E-F). These results suggest that a cellular response or cell communication-mediated cascade might occur at LD7 in the mediobasal hypothalamus.

**Fig. 6:**
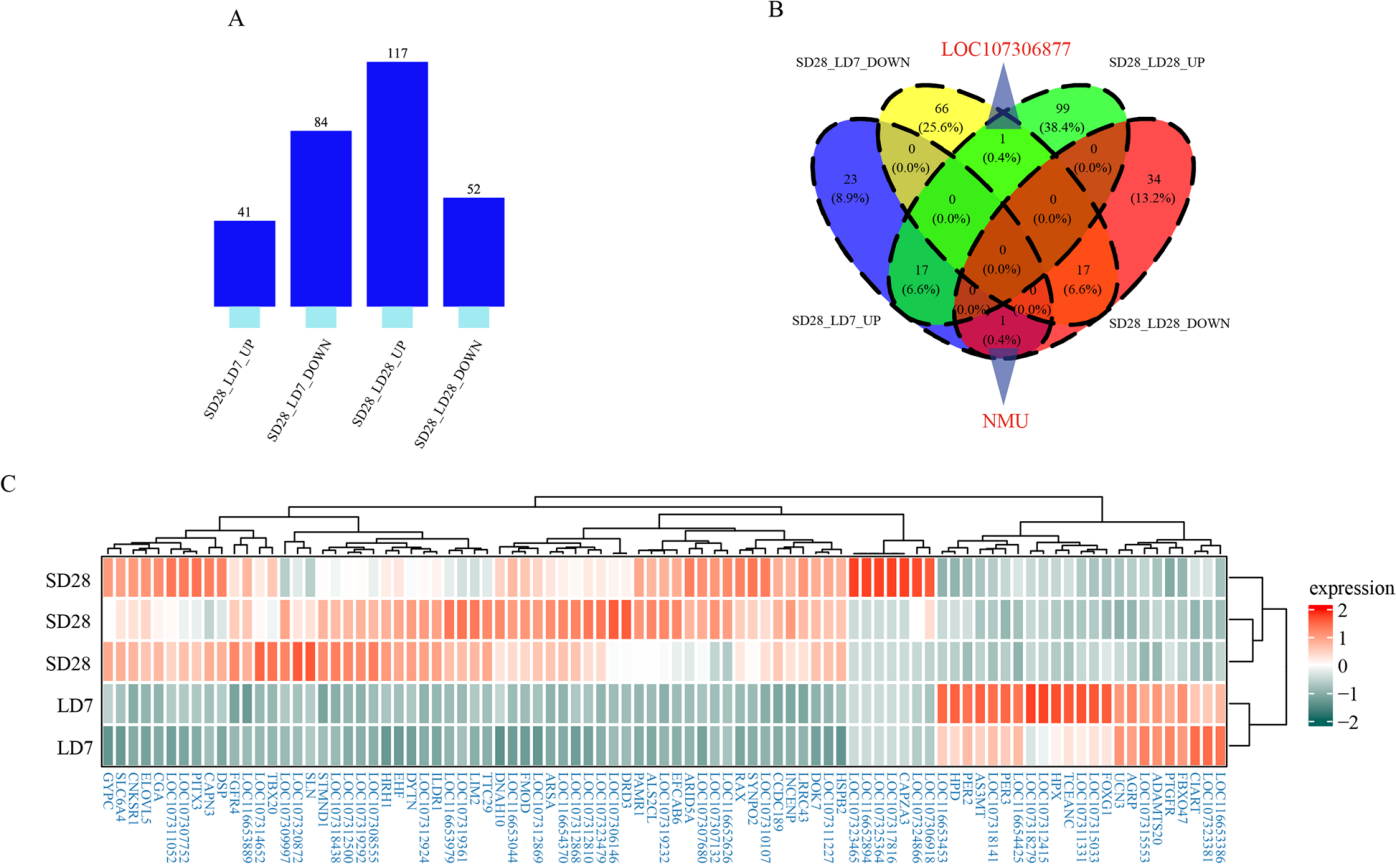
Identification of early light response genes combined with published transcriptome data. **A** The distribution of up-regulated and down-regulated genes in the two groups of difference analysis. **B** The overlap analysis of DEGs, in which LOC107306877 and NMU were marked. **C** The heatmap shows the expression of 89 early photoresponse genes between SD28 and LD7

To further identify the early photoperiod-dependent gene candidates, we downloaded published transcriptome expression data for the male Japanese quail hypothalamus at SD28 and LD28 [56] (Fig. 6A). Among the DEGs, 89 genes were unique to our transcriptome sequencing data; these gene included 23 upregulated DEGs (such as PER2, PER3, AGRP, and PTGFR) and 69 downregulated DEGs (such as CGA, RAX, and PITX3) (Fig. 6C). Thirty-six genes, including DIO2, DIO3, TSHB, and GHRH, were shared by two compared group, SD28 vs. LD7 and SD28 vs. LD28. A total of 133 genes, including PGR, RGR, RFX6, RXRG, were stage specific in SD28 vs. LD28. Interestingly, the expression of NMU (neuromedin U), a member of the neuropeptide neuromedin family, was upregulated at LD7 relative to SD28 (Fig. 6B). Moreover, LD28 was downregulated relative to SD28, suggesting that the gene was strongly regulated during the LD7-to-LD28 transition. In contrast to the NMU gene, LOC107306877 (inositol 1,4, 5-trisphosphate receptor-like 1) was deactivated at LD7 relative to SD28 (Fig. 6B). In summary, we identified several time-specific genes that may play regulatory and complex functions in response to photoperiod changes.

### Chromatin accessibility regulates gene expression in thyroid hormone signaling

**Fig. 7:**
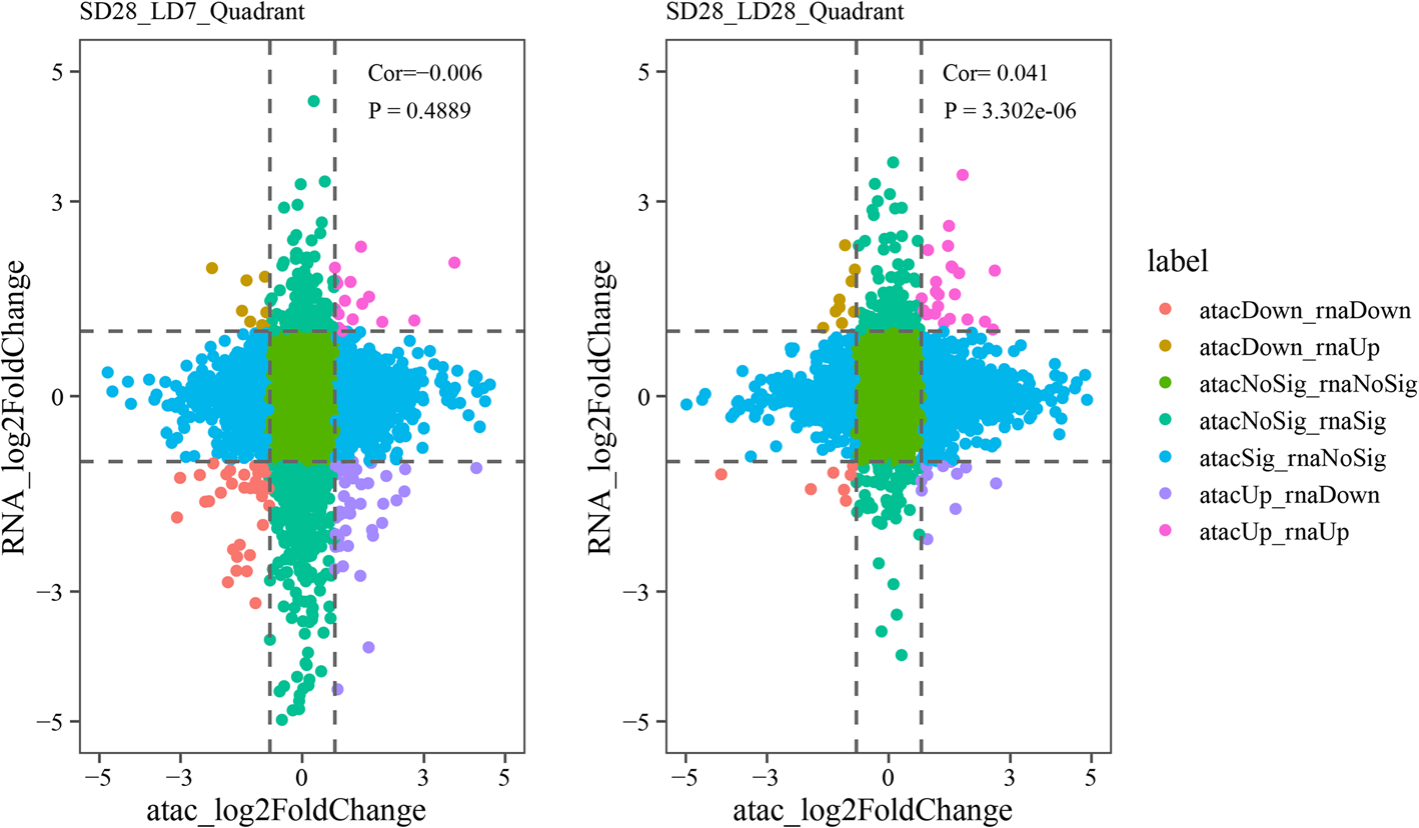
The nine-quadrant plots show the correlation of open chromatin and gene expression in the hypothalamus of male Japanese quail between SD28 and LD7, and between SD28 and LD28. The meaning of each quadrant is indicated by the label in the figure. All scatter points are assigned to seven types by definition, and the meaning of each type is represented by a label in the diagram

Photoperiod-response TFs might be activated prior to their downstream target genes through their interacting cis-regulatory elements. To determine the regulatory effect of chromatin accessibility on the expression of nearby genes during the seasonal activation of the HPG axis. We analyzed the correlation between DARs and DEGs in two comparison groups (SD28 vs. LD7 and SD28 vs. LD28) based on the genomic annotation of peaks present in the nine-quadrant plots. Our results showed that chromatin accessibility changes were significantly correlated with changes in expression in the SD28 vs. LD28 comparisons (Pearson correlation, r = 0.041, *P* = 3.302e^-06^) (Fig. 7B). However, no significant correlation was detected between SD28 and LD7 (Pearson correlation, r = − 0.006, *P* = 0.48) (Fig. 7A).

**Fig. 8:**
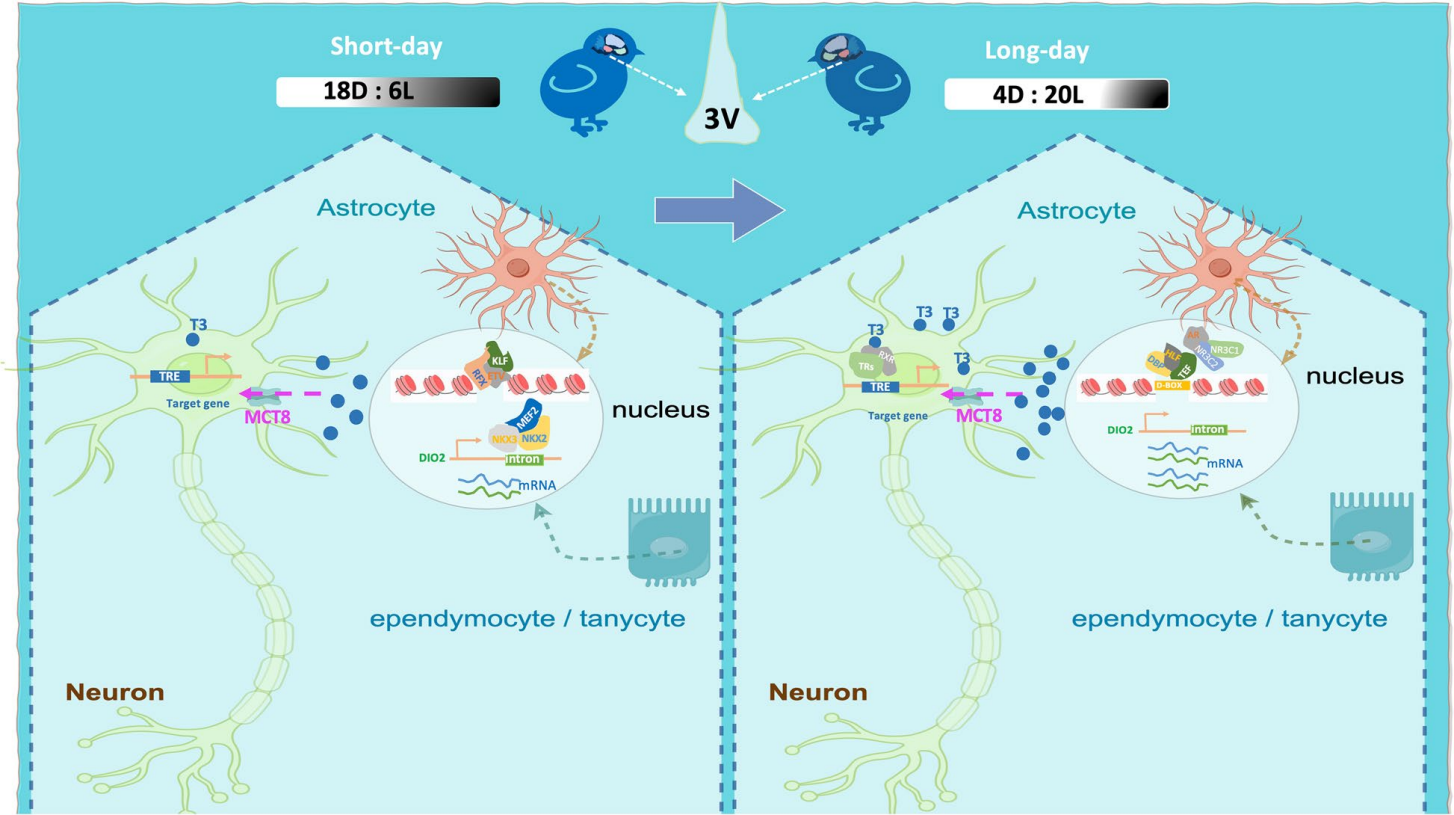
Summary of the chromatin changes in the MBH under short-day and long-day in quail. The model proposes changes in chromatin accessibility in the MBH region under the long- and short-day. DBP, TEF, HLF, and THRA were the first responders to long days. Chromatin accessibility shapes the different expression patterns of the DIO2 gene between the long- and the short-day. This model, in combination with previous studies [16, 57], showed the underlying genetic mechanism involved in the remodeling of the hypothalamic interface under the long- and the short-day

Gene Ontology analysis of 8 DEGs associated with the gained-loss chromatin accessibility region revealed enrichment of pathways related to thyroid hormone signaling, including PDE6H and DIO2 in LD7, and SLC6A2, and DIO2 in LD28. PDE6 is involved in visual transduction in photoreceptor cells and can be activated by a cascade of photons and opsins, and PDE6H plays a critical role in controlling the physiological adaptation of the photosensitive system to changes in the light environment. DIO2 was more highly expressed in LD7 and LD28 due to the loss of the binding site of seven transcription factors (Table 1). SLC16A2 encodes the high-affinity thyroid hormone T3- and T4- transporter (MCT8), which plays a decisive role in the transport of T3 into neurons. The expression of SLC16A2 was downregulated in LD28, possibly due to the involvement of transcriptional repressors in regulating gene expression. In brief, we summarized the changes in the MBH under long- and short-day conditions during the activation of the HPG axis and combined these finding with previous findings that reported the dynamic interaction of glial interfaces and neurons in quail (Fig. 8).

**Table 1:** | Loss of chromatin accessibility in long-day influence TF interaction in DIO2 gene

Transcription factors	Motif sequence	Enrichment Score	Strands	Location
Nkx2.5	TGAAGTGCAT	6.512154	–	Intron
Nkx2.1	TTGAAGTGCA	6.530204	–	Intron
Nkx2.2	TTGAAGTGCA	6.855058	+	Intron
Mef2c	GCTAAAATTAAC	8.212733	+	Intron
Mef2d	GCTAAAATTAAC	9.079877	–	Intron
Nkx3.2	TGAAGTGCAT	6.477036	+	Intron
Mef2b	GCTAAAATTAAC	7.830211	–	Intron

### Identification of THRB binding sites and their regulatory effects on gene expression

THRB is a crucial transcription factor involved in seasonal activation of the HPG axis. To gain further insight into how THRB regulates the expression of downstream HPG axis seasonal activation-response genes, we performed DNA affinity purification and sequencing (DAP-seq) using the THRB protein. After analyzing the sequenced reads, we identified 1665 and 2548 potential THRB binding sites from two biological replications, with an overlap of 1118 binding sites. The binding sites were annotated according to the genome annotation files and assigned to 728 genes. Approximately 8.41% (*n* = 94) of the identified consensus peaks were located in promoter regions, within 2 kb upstream and downstream of the TSSs. Approximately 5.73% of the identified consensus peaks were found in exons. Peaks were highly enriched in genome intronic regions.

To further examine whether THRB binding affects gene expression, we calculated the overlap between high-confidence binding sites and differentially expressed genes in the transcriptome. We found that the TRPA1 gene overlapped between the LD7 and SD28 at the early photoperiod transition and that THRB binds to the intron of TRPA1. We hypothesized that THRB promotes the expression of TRPA1 by binding to the enhancer sequence associated with it. With the same treatment but different, for genes that differed from LD28 and SD28, we found that the binding site affected more genes, including RASD2, PGR, TRPA1, SHROOM1, TRPM6FRMPD1, and LOC107307440.

Using meme software, we identified significantly distinct THRB binding motifs [T/C/A][G/A/C][A/G]GG[T/A]CA, the top 5 of which are shown in the Additional file 1. According to our DAP-seq data, THRB transcription factors were significantly enriched, and the binding distribution was highly concentrated, which further confirmed the ability of THRB to bind to the genome. GO term enrichment of the potential THRB targets indicated that they were associated mainly with protein binding (MF), signaling (BP), cell communication (BP), and membrane (CC). Furthermore, several KEGG pathways essential for signaling in the hypothalamus were also enriched, including the calcium signaling pathway and, phototransduction. Ca^2+^ concentration-mediated light adaptation mechanisms in the photoreceptor. Taken together, these findings imply that THRB plays an important role in regulating long-light stimulation mechanisms at various levels.

## Discussion

The photoperiod affects reproduction in seasonal breeders through the hypothalamic-pituitary-gonadal (HPG) axis, in which the mediobasal hypothalamus (MBH) is the central region that transmits light information to the endocrine system in birds. The gene expression landscape of the MBH is highly dynamic during reproductive inactivation under short-day condition and plays a fundamental role in regulating reproductive activation under long-day condition in quail [56]; however, the cis-regulatory elements and transcriptional activation mechanisms in MBH related to seasonal activation of the reproductive axis in the MBH remain largely unclear. The atlas of open chromatin enabled the exploration of gene regulation during the activation of the HPG axis in quail. In this study, we comprehensively profiled the change in chromatin accessibility in the MBH during the initiation of the reproductive axis activation in quail. The dynamic of chromatin accessibility was observed from the inactivation of the HPG axis to the initiation of the reproductive axis, and the active chromatin accessible region in the MBH region increased gradually in the enhancer region (intron and intergenic region) from the SD to the LD.

The alteration in chromatin accessibility status in the MBH was stabilized at LD7 during the activation of the HPG axis. In addition to genes associated with classic retinoic acid signaling, genes associated with gained chromatin opening were significantly enriched in pathways related to GTPase-mediated signal transduction. Small GTPases usually act as a molecular switches in regulating signal transduction [58] In the visual system, cGMP is the main intracellular messenger that converts light stimuli into electrical responses, and the balance of cGMP is regulated by the photoreceptor phosphodiesterase PDE6 [59], Activated PDE6 can mediate the closure of cyclic nucleotide-gated channels (CNG channels) by hydrolyzing cGMP, thus enabling the transmission of optical signals [60]. In addition, the transcriptional activity of PDE6H is regulated by T3 by binding to the TRs [7, 61, 62]. In vertebrate photoreceptors, a light-activated GTPase shows remarkable sensitivity to light, and plays a key role in the regulation of light-sensitive phosphodiesterase in vivo [63].

Motif analysis of DARs revealed a broad decrease in chromatin accessibility, in which the RFX, NKX, SOX, and MEF transcription factors families exhibited high chromatin accessibility under short-day conditions and gain-loss accessibility under long-day conditions. The members of the NKX families were significantly enriched in open DARs under short-day conditions. Nkx2.2 is a target of the sonic hedgehog pathway and a key regulator of the differentiation and maturation of oligodendrocytes in chicken brains [64]. NKX2.1, known as thyroid-specific-enhancer-binding protein (TTF-1), is involved in activating thyroid-specific gene expression [65]. NKX2.1 was initially found to regulate the transcription of the thyroid. Excessive retinoic acid (RA) overload is associated with brain abnormalities. Moreover, Nkx2.1 Knockout lead to a drastic loss of astrocytes [66] and a dramatic reduction in the size and severe malformation of the basal hypothalamus [67]. NKX2.5 is an essential cardiac development transcription factor, and mutations in NKX2.5 lead to impaired cardiac circulation and defects in gene expression associated with the myocardium [68]. These factors might contribute to fine-tuning the circadian clockwork in peripheral tissues [69]. NKX2.5 was the candidate gene for thyroid dysgenesis [70]. During the activation of the HPG axis, NKX2.1, NKX2.2, and NKX2.5 were identified as highly active under short-day condition and lost their binding site with a significant reduction in chromatin accessibility under long light exposure, suggesting that the NKX2 transcription factors family is likely involved in inhibiting the activation of the HPG axis.

After long-light exposure, binding motifs of both thyroid hormone-related and retinoid X receptor-related NR transcription factors families were significantly enriched in DARs which exhibited high chromatin accessibility under long-day conditions, with gain-loss of accessibility under short-day conditions. The binding motifs of Retinoic acid-related orphan receptor-γ (RORγt), Liver X receptor (LXRE), THRB, and THRA exhibited significantly high chromatin accessibility in LD7. Retinoic acid-related orphan receptors (RORs) have been implicated in controlling circadian rhythm [71, 72], and RORγt plays a regulatory role in Npas2-dependent physiological processes such as circadian behavior disorders [73]. Local T3 in the MBH of the hypothalamus controls the balance of reproduction and energy metabolism in the central nervous system [74]. An increase in T3 synthesis in the MBH leads to the synthesis and release of gonadotropin-releasing hormone (GnRH) and stimulates reproductive activity in long-day breeders [5]. Thyroid hormone (T3, T4) regulates gene expression predominantly mediated via the thyroid hormone receptors THRA and THRB; whether or not the THRs are bound to T3 can alter the transcription rates of target genes [75]. Previous studies have suggested the divergent physiological roles of THRA and THRB in physiology, and THRB is the master regulator of the negative feedback loop of the HPT axis that maintains circulating thyroid hormone levels [75].

The binding motifs of the steroid hormone-related NR superfamily (GRE, PGR, and ARE) were significantly enriched at the active chromatin regions in LD28, which functions as transcription factors and binds to chromatin to affect the expression level of downstream genes at the molecular level [76]. Steroid hormones can act as chemical messengers to produce both slow genomic responses and rapid nongenomic responses in target tissues [77]. Glucocorticoids induce lasting epigenetic modifications in many target tissues [78] and regulate the transcription of the glucocorticoid-responsive genes by binding to GREs [79, 80]. In addition, GR-regulated gene network transcription can be easily altered by ligands [81]. Progesterone is an obligatory transcriptional regulator that mediates ovulation signaling through the progesterone receptor (PGR) [82]. The PGR plays diverse role in reproductive traits and mediates the induction of essential ovulatory genes by chromatin remodeling in reproductive tissues [83, 84]. Androgen receptor (AR) dimers bind to androgen response elements (AREs) in the promoter regions of target genes in the nucleus and play a pivotal role in generating the male reproductive phenotype [85].

Further footprint analysis revealed that THRA had the most significant promoting effect on the initiation of the reproductive axis in LD3, and several circadian clock-output genes TEF, DBP, and HLF, were identified [86]. DBP, HLF, and TEF are three PAR bZip (proline and alkaline leucine zipper rich in acidic amino acids) transcription factors. All three transcriptional regulatory proteins accumulate with a robust circadian rhythm in tissues where clock genes are highly expressed [87]. Both the transcription factors DBP and TEF are essential elements of the “cell clock” [88]. Photoperiodic sensitivity is reduced by the TEF-dependent pathway [89]. The circadian accumulation of DBP and TEF in brain tissues is thought to be involved in controlling the circadian regulation of downstream genes. A high mRNA level of TEF was rapidly induced in hyperthyroidism [90]. Triiodothyronine plays a vital role in regulating pituitary gland homeostasis and can ultimately influence the rhythmic synthesis and/or secretion of hormones in the anterior pituitary. The TEF is considered a molecular switch for photoperiod responsiveness in mammals [86]. In the PT of mammals, long-day exposure rapidly induces expression of the coactivator eyes absent 3 (EYA3), which synergizes with TEF to maximize TSHβ transcription [32, 86]. In birds and other vertebrates, the TEF/Six1/Eya3-dependent mechanism will be the conserved driver of photoperiod induced changes in reproduction [86]. Circadian transcription of the clock-control gene DBP is driven by the transcription factors BMAL1 and CLOCK [91] and activates transcription of the Per1 gene by binding to the promoter region [92].

Considering the central control of thyroid function on the seasonal activation of the HPG axis, the transcription factors THRA and THRB may be the master regulators of reproductive initiation and maintenance processes when transitioning to long light exposure. However, the molecular interactome of THRs and the mechanism through which THRs induce genes in the MBH that are essential for the seasonal activation of the HPG axis are unknown—thyroid signaling cross-talk with retinoic acid pathways to stimulate SLC16A2 expression and thyroid transport in the brain [93]. At the molecular level, when modulating the expression of target genes, THRs bind to thyroid hormone response elements (TREs) located in regulatory regions of thyroid hormone response genes. When T3 bind to the THRs, THRs undergo chromatin conformational modification, which leads to activation or inhibition of the gene transcription machinery [94].

Chromatin accessibility is a critical regulatory factor influencing gene expression. The bioavailability of T3 in the MBH plays a crucial role in regulating the seasonal reproduction [95]. The correlation between differential chromatin accessibility and differentially expressed genes in our study further support the functional relevance of the thyroid hormone metabolism genes. DIO2 and SLC16A2 likely play significant roles in regulating the seasonal activation of the HPG axis in the MBH in quail. The photoperiodic control of the metabolism of tanycytes mainly depends on the expression of deiodinase (Dio2 and Dio3) to regulate the bioavailability of thyroid hormone in the local hypothalamus [96].SLC16A2 encodes a highly active and selective thyroid hormone transporter, MCT8, that facilitates the cellular uptake of thyroid hormones in different tissues. MCT8 deficiency disrupts the transfer of thyroid hormones across the blood brain barrier and can also lead to hypothyroidism [97].

We aimed to elucidate how chromatin accessibility shapes the expression pattern of the DIO2 gene during the seasonal activation of the HPG axis. According to our footprint analysis, NKX2.1, NKX2.2, and NKX2.5 lacked the binding site in the intron region of the DIO2 gene, which affected its expression under long-day conditions. It was suggested that the gene expression of DIO2 is generally regulated by both TFs in the NKX family and the Myocyte enhancer factor 2 (MEF2) family, which interact with cis-regulatory DNA elements. In humans, the DIO2 gene is stimulated by NKX2.1, and the NKX2.1 knockout mice were born dead without a thyroid or pituitary gland or defects in thyroid function [98]. MEF2 transcription factors are importance in the nervous system [99] and are considered effector of neurogenesis in the brain [100]. In the nervous system, MEF2 plays a central regulatory role in neuronal survival and axonal growth by controlling the expression of its target genes [101]. The MEF2C limits excessive synapse formation and regulates basal synaptic transmission, which is essential for facilitating learning and memory formation [102]. In the retina, MEF2D binds to retina-specific enhancers and controls photoreceptor cell development (cells Field 84). MEF2B and MEF2C are required for adhesion-related kinases to inhibit GnRH gene expression in GnRH neurons [103]. It was suggested that cis-acting elements in DIO2 and their relevant TFs are were the key players associated with HPG axis activation in long-light stimulation.

Thyroid hormones are essential for seasonal reproduction in vertebrates because of their apparent importance in GnRH release [26]. THRB mediates most of the actions of T3; however, the molecular regulatory mechanism through which THRB is regulated at the molecular level remains to be determined. Identifying the biosynthetic pathways and the target genes of THRB is essential for understanding the molecular regulatory mechanism involved in the seasonal activation of the HPG axis in quail. In our study, enrichment of the THRB motif in accessible chromatin occurred during the reproductive activation of the HPG axis. Eight THRB target genes exhibited differential expressions between short days and long days.

In LD7, the THRB-target gene transient receptor potential ankyrin 1 (TRPA1) exhibited differential expression. Transient receptor potential (TRP) ion channels are crucial for many senses, including touch, vision, and olfaction [104, 105]. The TRP gene is essential for a light-activated Ca^2+^ channels in photoreceptors of Drosaphila [106]. TRPA1 is a member of the TRP channel family and has the chemo-optogenetic property of high conductivity; TRPA1 exhibits higher conductance than does channelrhodopsin [107]. Optovin, a photochemical ligand of TRPA1, enables optical control of endogenous channels and neuronal signaling. Photodetection is performed by sensory neurons expressing the cation channel TRPA1 [108]. In zebrafish, the biological response to Trpa1b photo-activation was highly sensitive and was very rapid, with a delay in the production of motion on a millisecond scale from the introduction of light [109]. It was suggested that THRB-regulated TRPA1 plays an essential role in the seasonal activation of the HPG axis.

 In this study, we aimed to offer novel insights into the mechanisms underlying seasonal reproductive activation from the perspectives of chromatin accessibility and transcription factors. However, our study was carried out at the bulk level, and the MBH is a complex and heterogeneous area. This limitation made it impossible to establish the exact association between open chromatin and gene expression or to identify cell type-specific transcription factors. To gain deeper insights into the intricate regulatory mechanisms underlying seasonal reproduction, future investigations employing higher resolution omics techniques will be invaluable. These advanced approaches will enable us to unravel the role of epigenetic regulation in this process and shed light on the complex interplay between chromatin dynamics and gene expression.

## Conclusions

In summary, we investigated the changes in chromatin accessibility and gene expression in the MBH during the seasonal activation of male quails by combining ATAC-seq and RNA-seq We identified the active regulatory elements and transcription factors involved in regulating the photoperiodic response. Retinoic acid signaling and GTPase-mediated signal transduction are involved in adaptation to long days and maintenance of HPG axis activation. In addition, we analyzed the potential targets of the key transcription factor THRB during the seasonal activation by Dap-seq. Further analysis suggested that the trans effects were the main factor affecting gene expression. Our findings shed light on the molecular mechanism underlying seasonal reproductive activation and highlight the importance of chromatin accessibility and transcription factor regulation in this process.

### Supplementary Information

**Additional file 1: Fig. S1.** Number of insert length statistics for each ATAC-seq library. **Fig. S2**. Pearson corrolation analysis for each ATAC-seq library. **Fig. S3**. The number of peaks called by MACS2 in each ATAC-seq library, the dotted line represents the numerical value of the reproducible peaks under this treatment. **Fig. S4**. Peak density in Japanese quail's genome. The outer circle is the chromosome, followed by the gene density distribution, and then the peak density distribution of SD28, LD3, LD7, and LD28. Some regions with high gene density and high peak distribution are selected by the lavender rectangle box. **Fig. S5**. Top 30 transcription factors enriched on the common peak at different time points. (A) SD28. (B) LD3. (C) LD7. (D) LD28. **Fig S6**. The volcano plot shows the difference analysis of ATAC-seq at SD28, LD3, LD7, and LD28 time points by DESEQ2 package. with blue dots representing the up-regulated region and red dots representing the down-regulated region. **Fig. S7**. The number and distribution statistics of chromatin regional differences in ATAC-seq at different time points. **Fig. S8**. GO enrichment analysis for K-means clustering of differential chromatin regions. **Fig. S9**. Binding characteristics of THRB transcription factors in DAP-seq. DAP-seq analysis of Japanese quail THRB protein. **Fig. S10**. Gene ontology analysis in biology process (BP) of the target site of THRB. **Fig. S11**. Gene ontology analysis in cellular component (CC) of the target site of THRB. **Fig. S12**. Gene ontology analysis in molecular function (MF) of the target site of THRB.

**Additional file 2: Table S1**. The library information of ATAC-seq. **Table S2**. The library information of transcriptome sequencing. **Table S3**. The library information of DAP-seq. **Table S4**. Transcription factor enrichment analysis results at the genome-wide level at SD28. **Table S5**. Transcription factor enrichment analysis results at the genome-wide level at LD3. **Table S6**. Transcription factor enrichment analysis results at the genome-wide level at LD7. **Table S7**. Transcription factor enrichment analysis results at the genome-wide level at LD28. **Table S8**. Differential analysis of chromatin regions and region annotations in all the comparison. **Table S9**. The unique DEGs in the SD28 vs LD7 comparison. **Table S10**. The unique DEGs in the SD28 vs LD28 comparison. **Table S11**. The Common DEGs between two comparison SD28 vs LD7 and SD28 vs LD28. **Table S12**. Detection of an overlapping gene in the atacUP_rnaUP region through combined analysis of ATAC-seq and RNA-seq at LD7. **Table S13**. Detection of an overlapping gene in the atacUP_rnaUP region through combined analysis of ATAC-seq and RNA-seq at LD28.

## Data Availability

The datasets generated for this study can be found in the Sequence Read Archive (https://www.ncbi.nlm.nih.gov/sra) at NGDC, with the BioProject ID: PRJCA013237. GSA Accession Number for ATAC-seq data: CRA008910 https://ngdc.cncb.ac.cn/gsa/s/o4iRb1Jp GSA Accession Number for RNA-seq data: CRA008911 https://ngdc.cncb.ac.cn/gsa/s/Lc0j8p1J GSA Accession Number for DAP-seq data: CRA008912 https://ngdc.cncb.ac.cn/gsa/s/eL5BpX99

## References

[1] Follett BK, Davies DT, Gledhill B (1977). Photoperiodic control of reproduction in Japanese quail: changes in gonadotrophin secretion on the first day of induction and their pharmacological blockade. J Endocrinol.

[2] Pan JQ, Liufu S, Sun JF, Chen WJ, Ouyang HJ, Shen X, Jiang DL, Xu DN, Tian YB, He JH (2022). Long-day photoperiods affect expression of OPN5 and the TSH-DIO2/DIO3 pathway in Magang goose ganders. Poult Sci.

[3] Xu Y, Jiang D, Liu J, Fu Y, Song Y, Fan D, Huang X, Liufu S, Pan J, Ouyang H (2022). Photoperiodic changes in both hypothalamus neurotransmitters and circulating gonadal steroids Metabolomic profiles in relation to seasonal reproduction in male quail. Front Physiol.

[4] Karsch FJ, Bittman EL, Foster DL, Goodman RL, Legan SJ, Robinson JE (1984). Neuroendocrine Basis of Seasonal Reproduction. In: Proceedings of the 1983 Laurentian Hormone Conference. Edited by Greep RO, vol. 40.

[5] Yoshimura T (2013). Thyroid hormone and seasonal regulation of reproduction. Front Neuroendocrinol.

[6] Kuenzel WJ, Kang SW, Zhou ZJ (2015). Exploring avian deep-brain photoreceptors and their role in activating the neuroendocrine regulation of gonadal development. Poult Sci.

[7] Pérez JH, Tolla E, Bishop VR, Foster RG, Peirson SN, Dunn IC, Meddle SL, Stevenson TJ (2023). Functional inhibition of deep brain non-visual opsins facilitates acute long day induction of reproductive recrudescence in male Japanese quail. Horm Behav.

[8] Pérez JH, Tolla E, Dunn IC, Meddle SL, Stevenson TJ (2019). A comparative perspective on extra-retinal photoreception. Trends Endocrinol Metab.

[9] Yoshimura T, Yasuo S, Watanabe M, Iigo M, Yamamura T, Hirunagi K, Ebihara S (2003). Light-induced hormone conversion of T4 to T3 regulates photoperiodic response of gonads in birds. Nature.

[10] Wood S, Loudon A (2014). Clocks for all seasons: unwinding the roles and mechanisms of circadian and interval timers in the hypothalamus and pituitary. J Endocrinol.

[11] Garcia-Fernandez JM, Cernuda-Cernuda R, Davies WI, Rodgers J, Turton M, Peirson SN, Follett BK, Halford S, Hughes S, Hankins MW (2015). The hypothalamic photoreceptors regulating seasonal reproduction in birds: a prime role for VA opsin. Front Neuroendocrinol.

[12] Hanon EA, Lincoln GA, Fustin JM, Dardente H, Masson-Pévet M, Morgan PJ, Hazlerigg DG (2008). Ancestral TSH mechanism signals summer in a photoperiodic mammal. Curr Biol.

[13] Nakao N, Ono H, Yamamura T, Anraku T, Takagi T, Higashi K, Yasuo S, Katou Y, Kageyama S, Uno Y (2008). Thyrotrophin in the pars tuberalis triggers photoperiodic response. Nature.

[14] Yasuo S, Watanabe M, Nakao N, Takagi T, Follett BK, Ebihara S, Yoshimura T (2005). The reciprocal switching of two thyroid hormone-activating and -inactivating enzyme genes is involved in the photoperiodic gonadal response of Japanese quail. Endocrinology.

[15] Barrett P, Ebling FJ, Schuhler S, Wilson D, Ross AW, Warner A, Jethwa P, Boelen A, Visser TJ, Ozanne DM (2007). Hypothalamic thyroid hormone catabolism acts as a gatekeeper for the seasonal control of body weight and reproduction. Endocrinology.

[16] Wood SH, Christian HC, Miedzinska K, Saer BR, Johnson M, Paton B, Yu L, McNeilly J, Davis JR, McNeilly AS (2015). Binary switching of calendar cells in the pituitary defines the phase of the circannual cycle in mammals. Curr Biol.

[17] Sáenz de Miera C, Hanon EA, Dardente H, Birnie M, Simonneaux V, Lincoln GA (2013). Hazlerigg DG: circannual variation in thyroid hormone deiodinases in a short-day breeder. J Neuroendocrinol.

[18] Nakane Y, Yoshimura T (2014). Universality and diversity in the signal transduction pathway that regulates seasonal reproduction in vertebrates. Front Neurosci.

[19] Stevenson TJ, Lynch KS, Lamba P, Ball GF, Bernard DJ (2009). Cloning of gonadotropin-releasing hormone I complementary DNAs in songbirds facilitates dissection of mechanisms mediating seasonal changes in reproduction. Endocrinology.

[20] Stoney PN, Helfer G, Rodrigues D, Morgan PJ, McCaffery P (2016). Thyroid hormone activation of retinoic acid synthesis in hypothalamic tanycytes. Glia.

[21] Cho S, Chung J, Han J, Ju Lee B, Han Kim D, Rhee K, Kim K (2001). 9-cis-retinoic acid represses transcription of the gonadotropin-releasing hormone (GnRH) gene via proximal promoter region that is distinct from all-trans-retinoic acid response element. Brain Res Mol Brain Res.

[22] Altman R, Brutlag D, Karp P, Lathrop R, Searls D (1994). Proceedings: second international conference on intelligent systems for molecular biology. In*.* United States.

[23] Rodriguez E, Blazquez J, Pastor F, Pelaez B, Pena P, Peruzzo B, Amat P (2005). Hypothalamic Tanycytes: A Key Component of Brain–Endocrine Interaction. Int Rev Cytol.

[24] Rizzoti K, Lovell-Badge R (2017). Pivotal role of median eminence tanycytes for hypothalamic function and neurogenesis. Mol Cell Endocrinol.

[25] Prevot V (2002). Glial-neuronal-endothelial interactions are involved in the control of GnRH secretion. J Neuroendocrinol.

[26] Yamamura T, Hirunagi K, Ebihara S, Yoshimura T (2004). Seasonal morphological changes in the neuro-glial interaction between gonadotropin-releasing hormone nerve terminals and glial endfeet in Japanese quail. Endocrinology.

[27] Koopman ACM, Taziaux M, Bakker J. Age-related changes in the morphology of tanycytes in the human female infundibular nucleus/median eminence. J Neuroendocrinol. 2017;29(5) 10.1111/jne.12467 28295754

[28] Prevot V (2010). Plasticity of neuroendocrine systems. Eur J Neurosci.

[29] Stoney PN, Rodrigues D, Helfer G, Khatib T, Ashton A, Hay EA, Starr R, Kociszewska D, Morgan P, McCaffery P (2017). A seasonal switch in histone deacetylase gene expression in the hypothalamus and their capacity to modulate nuclear signaling pathways. Brain Behav Immun.

[30] Stevenson TJ, Prendergast BJ (2013). Reversible DNA methylation regulates seasonal photoperiodic time measurement. Proc Natl Acad Sci USA.

[31] Stevenson TJ (2018). Epigenetic regulation of biological rhythms: an evolutionary ancient molecular timer. Trends Genet.

[32] Wood SH, Hindle MM, Mizoro Y, Cheng Y, Saer BRC, Miedzinska K, Christian HC, Begley N, McNeilly J, McNeilly AS (2020). Circadian clock mechanism driving mammalian photoperiodism. Nat Commun.

[33] Dawson A, King VM, Bentley GE, Ball GF (2001). Photoperiodic control of seasonality in birds. J Biol Rhythm.

[34] Stevenson TJ. Environmental and hormonal regulation of epigenetic enzymes in the hypothalamus. J Neuroendocrinol. 2017;29(5) 10.1111/jne.12471 28370682

[35] Stevenson TJ (2017). Circannual and circadian rhythms of hypothalamic DNA methyltransferase and histone deacetylase expression in male Siberian hamsters (Phodopus sungorus). Gen Comp Endocrinol.

[36] Chen J, Bi H, Pettersson ME, Sato DX, Fuentes-Pardo AP, Mo C, Younis S, Wallerman O, Jern P, Molés G (2021). Functional differences between TSHR alleles associate with variation in spawning season in Atlantic herring. Commun Biol.

[37] Rubin CJ, Zody MC, Eriksson J, Meadows JR, Sherwood E, Webster MT, Jiang L, Ingman M, Sharpe T, Ka S (2010). Whole-genome resequencing reveals loci under selection during chicken domestication. Nature.

[38] Karlsson AC, Fallahshahroudi A, Johnsen H, Hagenblad J, Wright D, Andersson L, Jensen P (2016). A domestication related mutation in the thyroid stimulating hormone receptor gene (TSHR) modulates photoperiodic response and reproduction in chickens. Gen Comp Endocrinol.

[39] Corces MRTA, Hamilton EG, Greenside PG, Sinnott-Armstrong NA, Vesuna S, Satpathy AT, Rubin AJ, Montine KS, Wu B, Kathiria A, Cho SW, Mumbach MR, Carter AC, Kasowski M, Orloff LA, Risca VI, Kundaje A, Khavari PA, Montine TJ, Greenleaf WJ, Chang HY (2017). An improved ATAC-seq protocol reduces background and enables interrogation of frozen tissues. Nat Methods.

[40] Chen S, Zhou Y, Chen Y, Gu J (2018). Fastp: an ultra-fast all-in-one FASTQ preprocessor. Bioinformatics.

[41] Langmead B, Salzberg SL (2012). Fast gapped-read alignment with bowtie 2. Nat Methods.

[42] Li H, Handsaker B, Wysoker A, Fennell T, Ruan J, Homer N, Marth G, Abecasis G, Durbin R (2009). Genome project data processing S: the sequence alignment/map format and SAMtools. Bioinformatics.

[43] Institute B: Picard toolkit. Broad Institute*,* GitHub repository 2019.

[44] Quinlan AR, Hall IM (2010). BEDTools: a flexible suite of utilities for comparing genomic features. Bioinformatics.

[45] Ramirez F, Ryan DP, Gruning B, Bhardwaj V, Kilpert F, Richter AS, Heyne S, Dundar F, Manke T (2016). deepTools2: a next generation web server for deep-sequencing data analysis. Nucleic Acids Res.

[46] Liu T (2014). Use model-based analysis of ChIP-Seq (MACS) to analyze short reads generated by sequencing protein-DNA interactions in embryonic stem cells. Methods Mol Biol.

[47] Neph S, Kuehn MS, Reynolds AP, Haugen E, Thurman RE, Johnson AK, Rynes E, Maurano MT, Vierstra J, Thomas S (2012). BEDOPS: high-performance genomic feature operations. Bioinformatics.

[48] Love MI, Huber W, Anders S (2014). Moderated estimation of fold change and dispersion for RNA-seq data with DESeq2. Genome Biol.

[49] Zhou Y, Zhou B, Pache L, Chang M, Khodabakhshi AH, Tanaseichuk O, Benner C, Chanda SK (2019). Metascape provides a biologist-oriented resource for the analysis of systems-level datasets. Nat Commun.

[50] Heinz S, Benner C, Spann N, Bertolino E, Lin YC, Laslo P, Cheng JX, Murre C, Singh H, Glass CK (2010). Simple combinations of lineage-determining transcription factors prime cis-regulatory elements required for macrophage and B cell identities. Mol Cell.

[51] Yu G, Wang LG, He QY (2015). ChIPseeker: an R/Bioconductor package for ChIP peak annotation, comparison and visualization. Bioinformatics.

[52] Thorvaldsdottir H, Robinson JT, Mesirov JP (2013). Integrative genomics viewer (IGV): high-performance genomics data visualization and exploration. Brief Bioinform.

[53] Dobin A, Davis CA, Schlesinger F, Drenkow J, Zaleski C, Jha S, Batut P, Chaisson M, Gingeras TR (2013). STAR: ultrafast universal RNA-seq aligner. Bioinformatics.

[54] Liao Y, Smyth GK, Shi W (2014). featureCounts: an efficient general purpose program for assigning sequence reads to genomic features. Bioinformatics.

[55] Duittoz AH, Tillet Y, Geller S (2022). The great migration: how glial cells could regulate GnRH neuron development and shape adult reproductive life. J Chem Neuroanat.

[56] Morris KM, Hindle MM, Boitard S, Burt DW, Danner AF, Eory L, Forrest HL, Gourichon D, Gros J, Hillier LW (2020). The quail genome: insights into social behaviour, seasonal biology and infectious disease response. BMC Biol.

[57] Shinomiya A, Shimmura T, Nishiwaki-Ohkawa T, Yoshimura T (2014). Regulation of seasonal reproduction by hypothalamic activation of thyroid hormone. Front Endocrinol (Lausanne).

[58] Wennerberg K, Rossman KL, Der CJ (2005). The Ras superfamily at a glance. J Cell Sci.

[59] Cote RH (2021). Photoreceptor phosphodiesterase (PDE6): activation and inactivation mechanisms during visual transduction in rods and cones. Pflugers Arch - Eur J Physiol.

[60] Turunen T, Koskelainen A (2021). Functional modulation of phosphodiesterase-6 by calcium in mouse rod photoreceptors. Sci Rep.

[61] Cheng Y, Xu J, Fu Y, He N (2020). Expression and regulation of pde6h by thyroid hormone during metamorphosis in Paralichthys olivaceus. Front Physiol.

[62] Nakane Y, Shimmura T, Abe H, Yoshimura T (2014). Intrinsic photosensitivity of a deep brain photoreceptor. Curr Biol.

[63] Wheeler GL, Matuo Y, Bitensky MW (1977). Light-activated GTPase in vertebrate photoreceptors. Nature.

[64] Qi Y, Cai J, Wu Y, Wu R, Lee J, Fu H, Rao M, Sussel L, Rubenstein J, Qiu M (2001). Control of oligodendrocyte differentiation by the Nkx2.2 homeodomain transcription factor. Development.

[65] Lazzaro D, Price M, de Felice M, Di Lauro R (1991). The transcription factor TTF-1 is expressed at the onset of thyroid and lung morphogenesis and in restricted regions of the foetal brain. Development.

[66] Minocha S, Valloton D, Arsenijevic Y, Cardinaux J-R, Guidi R, Hornung J-P, Lebrand C (2017). Nkx2.1 regulates the generation of telencephalic astrocytes during embryonic development. Sci Rep.

[67] van den Akker WM, Brox A, Puelles L, Durston AJ, Medina L (2008). Comparative functional analysis provides evidence for a crucial role for the homeobox gene Nkx2.1/Titf-1 in forebrain evolution. J Comp Neurol.

[68] Jamali M, Rogerson PJ, Wilton S, Skerjanc IS (2001). Nkx2–5 activity is essential for Cardiomyogenesis. J Biol Chem.

[69] Bozek K, Relógio A, Kielbasa SM, Heine M, Dame C, Kramer A, Herzel H (2009). Regulation of clock-controlled genes in mammals. PLoS One.

[70] Cerqueira TLO, Ramos YR, Strappa GB, Jesus MS, Santos JG, Sousa C, Carvalho G, Fernandes V, Boa-Sorte N, Amorim T (2018). Mutation screening in the genes PAX-8, NKX2-5, TSH-R, HES-1 in cohort of 63 Brazilian children with thyroid dysgenesis. Arch Endocrinol Metab.

[71] Ueda HR, Hayashi S, Chen W, Sano M, Machida M, Shigeyoshi Y, Iino M, Hashimoto S (2005). System-level identification of transcriptional circuits underlying mammalian circadian clocks. Nat Genet.

[72] Jetten AM (2009). Retinoid-related orphan receptors (RORs): critical roles in development, immunity, circadian rhythm, and cellular metabolism. Nucl Recept Signal.

[73] Takeda Y, Kang HS, Angers M, Jetten AM (2011). Retinoic acid-related orphan receptor γ directly regulates neuronal PAS domain protein 2 transcription in vivo. Nucleic Acids Res.

[74] Helfer G, Barrett P, Morgan PJ (2019). A unifying hypothesis for control of body weight and reproduction in seasonally breeding mammals. J Neuroendocrinol.

[75] Ortiga-Carvalho TM, Sidhaye AR, Wondisford FE (2014). Thyroid hormone receptors and resistance to thyroid hormone disorders. Nat Rev Endocrinol.

[76] Margolis RN, Christakos S (2010). The nuclear receptor superfamily of steroid hormones and vitamin D gene regulation. An update. Ann N Y Acad Sci.

[77] Norman AW, Mizwicki MT, Norman DPG (2004). Steroid-hormone rapid actions, membrane receptors and a conformational ensemble model. Nat Rev Drug Discov.

[78] Zannas AS, Chrousos GP (2017). Epigenetic programming by stress and glucocorticoids along the human lifespan. Mol Psychiatry.

[79] Barnes PJ (1998). Anti-inflammatory actions of glucocorticoids: molecular mechanisms. Clin Sci (Lond).

[80] Johnson TA, Paakinaho V, Kim S, Hager GL, Presman DM (2021). Genome-wide binding potential and regulatory activity of the glucocorticoid receptor’s monomeric and dimeric forms. Nat Commun.

[81] Weikum ER, Knuesel MT, Ortlund EA, Yamamoto KR (2017). Glucocorticoid receptor control of transcription: precision and plasticity via allostery. Nat Rev Mol Cell Biol.

[82] Conneely OM (2010). Progesterone receptors and ovulation. Handb Exp Pharmacol.

[83] Dinh DT, Breen J, Nicol B, Foot NJ, Bersten DC, Emery A, Smith KM, Wong YY, Barry SC, Yao HHC (2023). Progesterone receptor mediates ovulatory transcription through RUNX transcription factor interactions and chromatin remodelling. Nucleic Acids Res.

[84] Dinh DT, Breen J, Akison LK, DeMayo FJ, Brown HM, Robker RL, Russell DL (2019). Tissue-specific progesterone receptor-chromatin binding and the regulation of progesterone-dependent gene expression. Sci Rep.

[85] Tan MHE, Li J, Xu HE, Melcher K (2015). Yong E-l: androgen receptor: structure, role in prostate cancer and drug discovery. Acta Pharmacol Sin.

[86] Dardente H, Wyse CA, Birnie MJ, Dupré SM, Loudon AS, Lincoln GA, Hazlerigg DG (2010). A molecular switch for photoperiod responsiveness in mammals. Curr Biol.

[87] Gachon F, Fonjallaz P, Damiola F, Gos P, Kodama T, Zakany J, Duboule D, Petit B, Tafti M, Schibler U (2004). The loss of circadian PAR bZip transcription factors results in epilepsy. Genes Dev.

[88] Allaman-Pillet N, Roduit R, Oberson A, Abdelli S, Ruiz J, Beckmann JS, Schorderet DF, Bonny C (2004). Circadian regulation of islet genes involved in insulin production and secretion. Mol Cell Endocrinol.

[89] Wehr TA (2001). Photoperiodism in humans and other primates: evidence and implications. J Biol Rhythm.

[90] Bargi-Souza P, Peliciari-Garcia RA, Nunes MT (2019). Disruption of the pituitary circadian clock induced by hypothyroidism and hyperthyroidism: consequences on daily pituitary hormone expression profiles. Thyroid.

[91] Stratmann M, Suter DM, Molina N, Naef F, Schibler U (2012). Circadian Dbp transcription relies on highly dynamic BMAL1-CLOCK interaction with E boxes and requires the proteasome. Mol Cell.

[92] Yamaguchi S, Mitsui S, Yan L, Yagita K, Miyake S, Okamura H (2000). Role of DBP in the circadian oscillatory mechanism. Mol Cell Biol.

[93] Kogai T, Liu YY, Richter LL, Mody K, Kagechika H, Brent GA (2010). Retinoic acid induces expression of the thyroid hormone transporter, monocarboxylate transporter 8 (Mct8). J Biol Chem.

[94] Brent GA (2012). Mechanisms of thyroid hormone action. J Clin Invest.

[95] Kampf-Lassin A, Prendergast BJ (2013). Acute downregulation of type II and type III iodothyronine deiodinases by photoperiod in peripubertal male and female Siberian hamsters. Gen Comp Endocrinol.

[96] Tu HM, Kim SW, Salvatore D, Bartha T, Legradi G, Larsen PR, Lechan RM (1997). Regional distribution of type 2 thyroxine deiodinase messenger ribonucleic acid in rat hypothalamus and pituitary and its regulation by thyroid hormone. Endocrinology.

[97] Bernal J, Guadaño-Ferraz A, Morte B (2015). Thyroid hormone transporters—functions and clinical implications. Nat Rev Endocrinol.

[98] Bz G (2001). Salvatore D, Harney JW, Tu HM, Larsen PR: the human, but not rat, dio2 gene is stimulated by thyroid transcription Factor-1 (TTF-1). Mol Endocrinol.

[99] Lisek M, Przybyszewski O, Zylinska L, Guo F, Boczek T (2023). The role of MEF2 transcription factor family in neuronal survival and degeneration. Int J Mol Sci.

[100] Li H, Radford JC, Ragusa MJ, Shea KL, McKercher SR, Zaremba JD, Soussou W, Nie Z, Kang YJ, Nakanishi N (2008). Transcription factor MEF2C influences neural stem/progenitor cell differentiation and maturation in vivo. Proc Natl Acad Sci USA.

[101] Mohajeri K, Yadav R, D'Haene E, Boone PM, Erdin S, Gao D, Moyses-Oliveira M, Bhavsar R, Currall BB, O'Keefe K (2022). Transcriptional and functional consequences of alterations to MEF2C and its topological organization in neuronal models. Am J Hum Genet.

[102] Barbosa AC, Kim MS, Ertunc M, Adachi M, Nelson ED, McAnally J, Richardson JA, Kavalali ET, Monteggia LM, Bassel-Duby R (2008). MEF2C, a transcription factor that facilitates learning and memory by negative regulation of synapse numbers and function. Proc Natl Acad Sci USA.

[103] Allen MP, Xu M, Zeng C, Tobet SA, Wierman ME (2000). Myocyte enhancer factors-2B and -2C are required for adhesion related kinase repression of neuronal gonadotropin releasing hormone gene expression. J Biol Chem.

[104] Damann N, Voets T, Nilius B (2008). TRPs in our senses. Curr Biol.

[105] Li T, Saito CT, Hikitsuchi T, Inoguchi Y, Mitsuishi H, Saito S, Tominaga M (2019). Diverse sensitivities of TRPA1 from different mosquito species to thermal and chemical stimuli. Sci Rep.

[106] Hardie RC, Minke B (1992). The trp gene is essential for a light-activated Ca2+ channel in Drosophila photoreceptors. Neuron.

[107] Pulver SR, Pashkovski SL, Hornstein NJ, Garrity PA, Griffith LC (2009). Temporal dynamics of neuronal activation by Channelrhodopsin-2 and TRPA1 determine behavioral output in Drosophila larvae. J Neurophysiol.

[108] Mourot A, Fehrentz T, Le Feuvre Y, Smith CM, Herold C, Dalkara D, Nagy F, Trauner D, Kramer RH (2012). Rapid optical control of nociception with an ion-channel photoswitch. Nat Methods.

[109] Lam P-Y, Mendu SK, Mills RW, Zheng B, Padilla H, Milan DJ, Desai BN, Peterson RT (2017). A high-conductance chemo-optogenetic system based on the vertebrate channel Trpa1b. Sci Rep.

