# Supplementary figures and images for: The dynamic landscape of chromatin accessibility and active regulatory elements in the mediobasal hypothalamus influences the seasonal activation of the reproductive axis in the male quail under long light exposure
Source: BMC Genomics. 2024 Feb 19;25:197. doi: 10.1186/s12864-024-10097-5 (PMC10877898; doi:10.1186/s12864-024-10097-5)

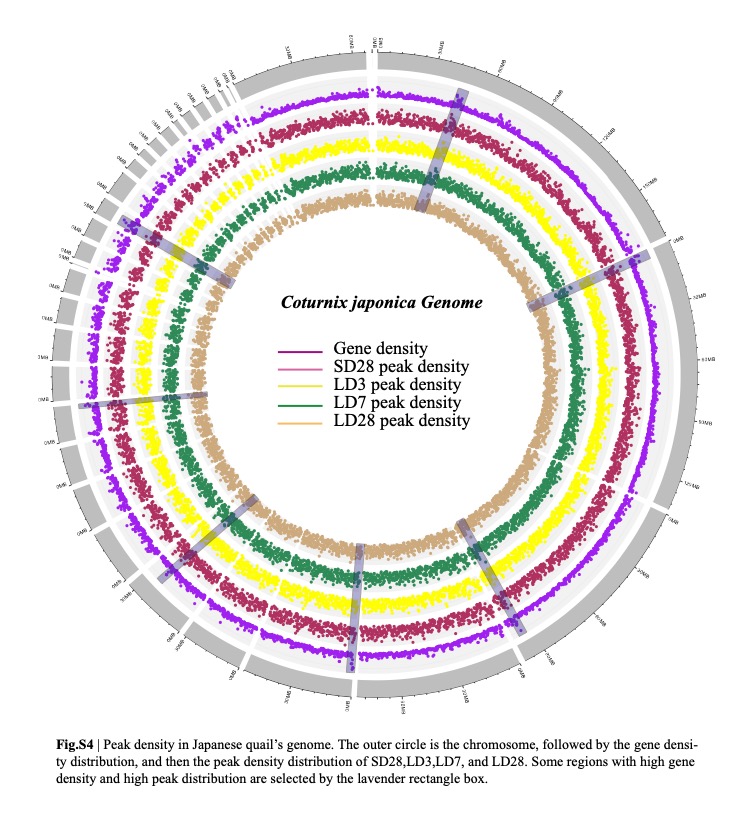


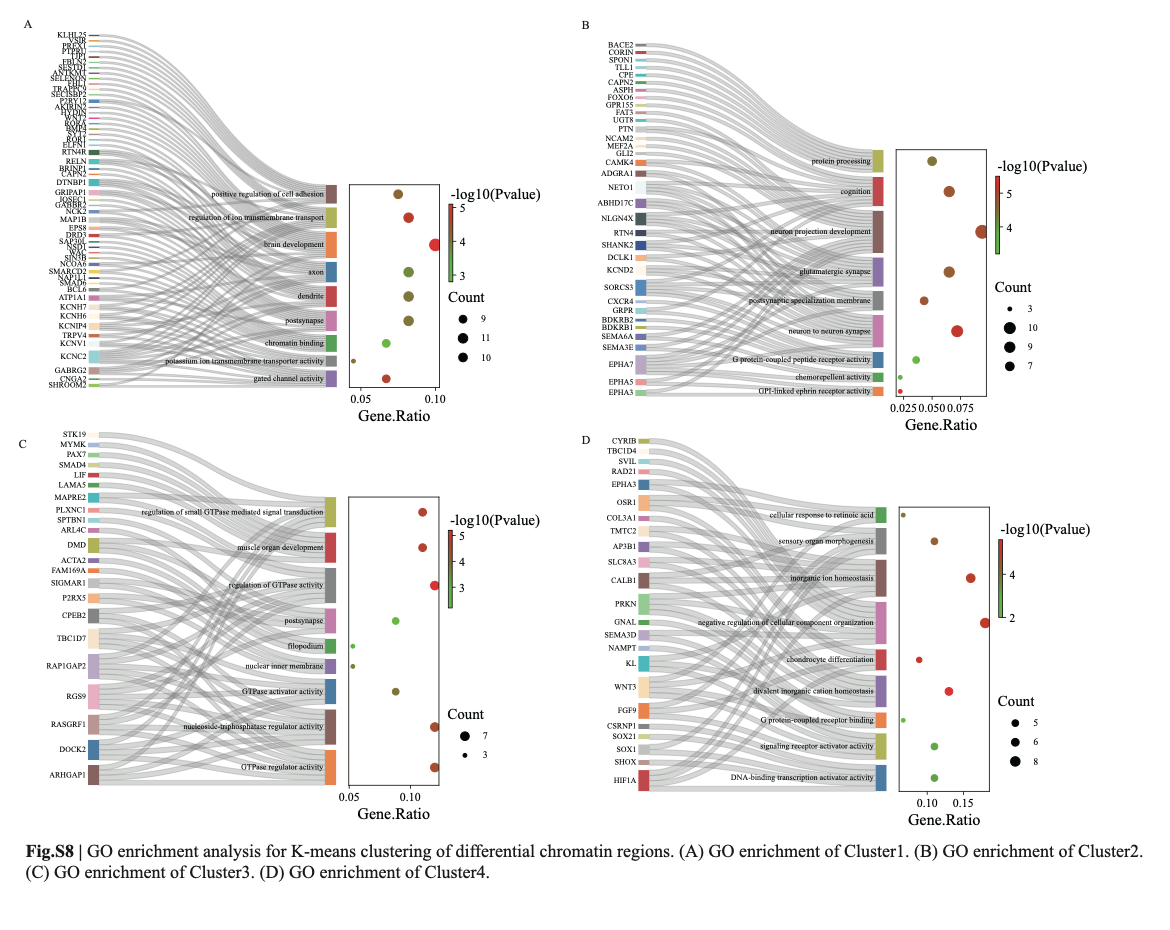


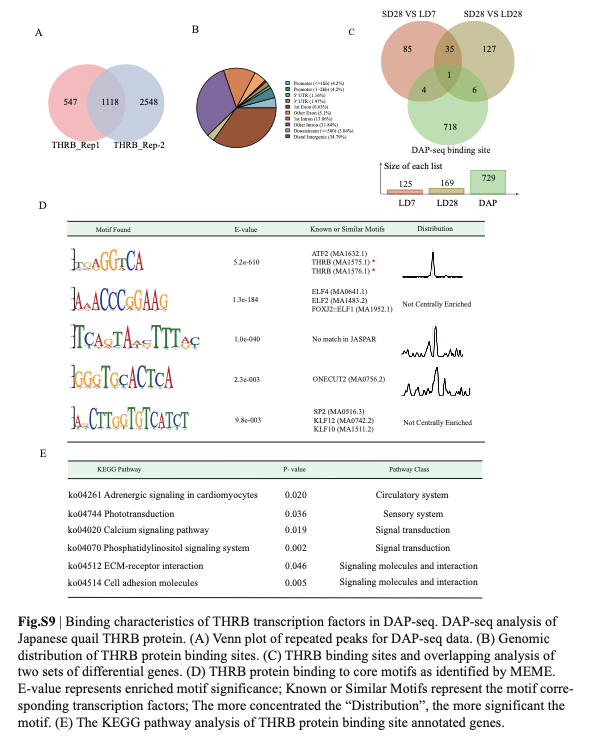

Supplement: Supplementary file 1 — Additional file 1: Fig. S1. Number of insert length statistics for each ATAC-seq library. Fig. S2. Pearson corrolation analysis for each ATAC-seq library. Fig. S3. The number of peaks called by MACS2 in each ATAC-seq library, the dotted line represents the numerical value of the reproducible peaks under this treatment. Fig. S4. Peak density in Japanese quail's genome. The outer circle is the chromosome, followed by the gene density distribution, and then the peak density distribution of SD28, LD3, LD7, and LD28. Some regions with high gene density and high peak distribution are selected by the lavender rectangle box. Fig. S5. Top 30 transcription factors enriched on the common peak at different time points. (A) SD28. (B) LD3. (C) LD7. (D) LD28. Fig S6. The volcano plot shows the difference analysis of ATAC-seq at SD28, LD3, LD7, and LD28 time points by DESEQ2 package. with blue dots representing the up-regulated region and red dots representing the down-regulated region. Fig. S7. The number and distribution statistics of chromatin regional differences in ATAC-seq at different time points. Fig. S8. GO enrichment analysis for K-means clustering of differential chromatin regions. Fig. S9. Binding characteristics of THRB transcription factors in DAP-seq. DAP-seq analysis of Japanese quail THRB protein. Fig. S10. Gene ontology analysis in biology process (BP) of the target site of THRB. Fig. S11. Gene ontology analysis in cellular component (CC) of the target site of THRB. Fig. S12. Gene ontology analysis in molecular function (MF) of the target site of THRB. [file 12864_2024_10097_MOESM1_ESM.docx]
